# 2’-Hydroxyflavanone effectively targets RLIP76-mediated drug transport and regulates critical signaling networks in breast cancer

**DOI:** 10.18632/oncotarget.24720

**Published:** 2018-04-06

**Authors:** Lokesh Dalasanur Nagaprashantha, Jyotsana Singhal, Hongzhi Li, Charles Warden, Xueli Liu, David Horne, Sanjay Awasthi, Ravi Salgia, Sharad S. Singhal

**Affiliations:** ^1^ Department of Medical Oncology, Beckman Research Institute of City of Hope, Comprehensive Cancer Center and National Medical Center, Duarte, CA 91010, USA; ^2^ Department of Molecular Medicine, Beckman Research Institute of City of Hope, Comprehensive Cancer Center and National Medical Center, Duarte, CA 91010, USA; ^3^ Department of Computational Therapeutics, Beckman Research Institute of City of Hope, Comprehensive Cancer Center and National Medical Center, Duarte, CA 91010, USA; ^4^ Department of Genomic Core, Beckman Research Institute of City of Hope, Comprehensive Cancer Center and National Medical Center, Duarte, CA 91010, USA; ^5^ Department of Information Sciences & Biostatistics, Beckman Research Institute of City of Hope, Comprehensive Cancer Center and National Medical Center, Duarte, CA 91010, USA; ^6^ Department of Internal Medicine, Texas Tech University Health Sciences Center, Lubbock, TX 79430, USA

**Keywords:** breast cancer, 2'-hydroxyflavanone, ERα, RLIP76, HER2

## Abstract

Breast cancer (BC) is the most common cancer in women. Estrogen, epidermal growth factor receptor 2 (ERBB2, HER2), and oxidative stress represent critical mechanistic nodes associated with BC. RLIP76 is a major mercapturic acid pathway transporter whose expression is increased in BC. In the quest of a novel molecule with chemopreventive and chemotherapeutic potential, we evaluated the effects of 2'-Hydroxyflavanone (2HF) in BC. 2HF enhanced the inhibitory effects of RLIP76 depletion and also inhibited RLIP76-mediated doxorubicin transport in BC cells. RNA-sequencing revealed that 2HF induces strong reversal of the gene expression pattern in ER^+^MCF7, HER2^+^ SKBR3 and triple-negative MDA-MB-231 BC cells with minimal effects on MCF10A normal breast epithelial cells. 2HF down regulated ERα and enhanced inhibitory effects of imatinib mesylate/Gleevec in MCF7 cells. 2HF also down regulated ERα and HER2 gene networks in MCF7 and SKBR3 cells, respectively. 2HF activated TP53 and inhibited TGFβ1 canonical pathway in MCF7 and MDA-MB-231 BC cells. 2HF also regulated the expression of a number of critical prognostic genes of MammaPrint panel and their upstream targets including TP53, CDKN2A and MYC. The collective findings from this study provide a comprehensive, direct and integrated evidence for the benefits of 2HF in targeting major and clinically relevant mechanistic regulators of BC.

## INTRODUCTION

Breast cancer (BC) remains the most common malignancy in women in spite of rapid advances in the methods for early detection and multi-modality interventions [1]. The interplay between the estrogen receptor (ER), epidermal growth factor receptor 2 (ERBB2 or HER2/neu), cellular proliferative and apoptotic networks collectively influence BC incidence and response to therapy [2]. The action of estrogen represents an endogenous chronic carcinogenic stimulus that determines both disease aggressiveness and prognosis in BC [3]. Amplification of HER2 is another major factor associated with both breast tumor response to clinical interventions and recurrence risk [4]. Mercapturic acid pathway (MAP) determines the intracellular levels of toxic lipid peroxidation products, tumor survival, chemo- and radio-sensitivity [5]. In MAP, RLIP76 (a ral-interacting protein; RalBP1) functions as a stress-responsive and ATP-dependent glutathione-electrophile conjugate (GS-E) transporter of toxic lipid peroxidation products and chemotherapy drugs [6]. Along with being a critical factor regulating clathrin-dependent endocytosis (CDE), RLIP76 is strongly linked to resistance to chemical as well as radiant stresses [7]. RLIP76 depletion does not affect the survival of normal cells while causing effective regression of multiple organ tumors including lung, colon, kidneys, skin, and pancreas [8–10]. RLIP76 is over-expressed in human BC making it a novel candidate for BC interventions [11].

Treatment of localized BC with surgery, radiation, chemotherapy and hormonal therapy is insufficient to control and eradicate BC as evident from the development of metastatic BC in up to 40% of women initially presenting with localized disease [12]. Natural phytochemicals have been a major source of both preventive and therapeutic lead compounds. More than >60% of the FDA approved drugs have been characterized and further developed from natural sources [13]. Citrus phytochemicals have sustained a strong focus of developmental research in the area of cancer prevention and/or therapy [14]. The citrus phytochemical 2'-Hydroxyflavanone (2HF) has shown promising efficacy against breast cancer [15]. According to Lipinski Rule of 5, for a drug/test compound to be orally active in humans, the total polar surface area should not be greater than 140 Ǻ ^2^, molar refractivity should be between 40 and 130, molecular weight should be less than 500, hydrogen bond donors should not be more than 5, hydrogen bond acceptors should not be more than 10 and partition coefficient (logP) should not be greater than 5 [16]. 2HF [Canonical SMILES: C1C (OC2=CC=CC=C2C1=O) C3=CC=CC=C3O] satisfies all the criteria of Lipinski Rule of 5 which makes it an ideal compound for regular use. Low total polar surface area of 2HF (TPSA, TPSA of 2HF-46.53) is a significant factor that could potentially facilitate better membrane penetration and permeability (Table 1) [17]. Hence, we investigated the efficacy of 2HF in regulating RLIP76 transport, docking of 2HF with RLIP76, ERα and HER2 along with elucidating the 2HF induced regulation of gene expression in BC.

**Table 1:** 2HF characteristics as a small molecule drug candidate according to Lipinski Rule of 5

Canonical Smiles	TPSA	MR	Molecular Weight	HBD	HBA	Log P	Lipinski Rule of 5
C1C(OC2=CC=CC=C2C1=O)C3=CC=CC=C3O	46.53	67.5245	240.25398	1	3	3.0987	100%

HBA-Hydrogen Bond Acceptors; HBD-Hydrogen Bond Donors; logP- Partition Coefficient; MR- Molar Refractivity; TPSA-Total Polar Surface Area.

Source: TargetNet- http://targetnet.scbdd.com/calcnet/index_rule/

## RESULTS

### Analyses of the effect of 2HF, alone and in combination with RLIP76 inhibition, on BC survival and ^14^C-Doxorubicin (^14^C-DOX) transport

**Figure 1:**
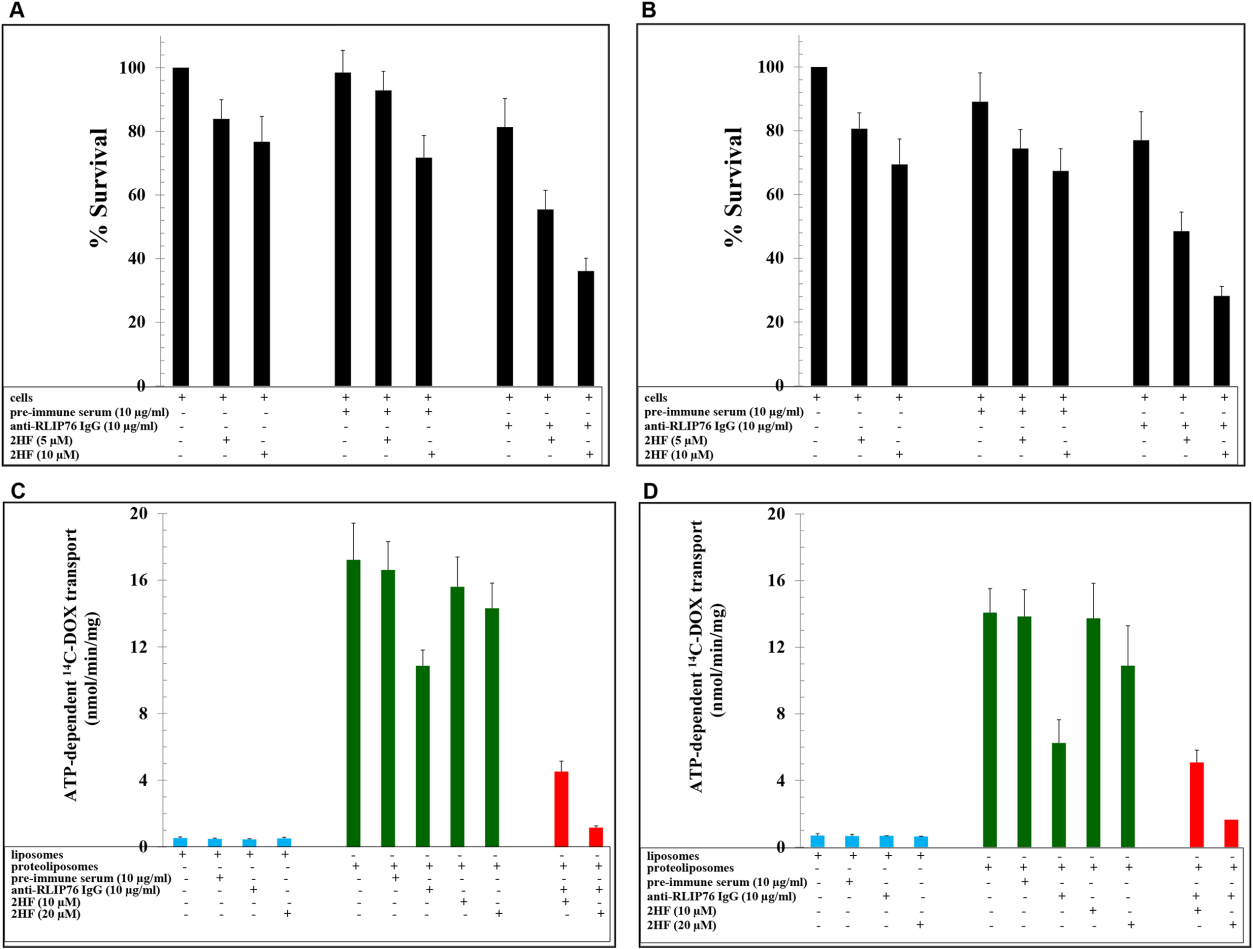
Effect of 2HF alone and in combination with RLIP76 inhibition in breast cancer survival and drug-resistance **(A-B)** The effects of anti-RLIP76 IgG (10 μg/ml conc.) and 2HF (5 and 10 μM conc.) and their combination on the survival of MCF7 (A) and MDA-MB-231 (B) cells conducted using MTT assay. **(C-D)** Effects of anti-RLIP76 IgG (10 μg/ml) and 2HF (10 and 20 μM) and their combination on the transport of ^14^C-DOX in purified reconstituted vesicles prepared from MCF7 (C) and MDA-MB-231 (D) cells performed by rapid-filtration technique using 250 ng RLIP76 protein per 30 μl reaction mixture.

RLIP76 is a multi-functional regulator of the cellular levels of oxidative stress and lipid peroxidation products and intracellular concentration of chemotherapy drugs like DOX, which is widely used in BC therapy [6–10]. 2HF at 5 and 10 μM concentrations led to inhibition of the survival of BC cells (MCF7: 16 ± 4 % and 23 ± 4 % at 5 and 10 μM 2HF, respectively; MDA-MB-231: 19 ± 4 % and 31 ± 6 % at 5 and 10 μM 2HF, respectively) and the addition of anti-RLIP76 IgG (10 μg/ml conc.) led to a stronger inhibition of BC cell survival (45 ± 6 % and 64 ± 6 % in MCF7 cells, and 52 ± 5 % and 72 ± 7 % in MDA-MB-231 cells, at 5 and 10 μM 2HF concentrations, respectively) (Figure 1A and 1B). DOX is a common drug transported by RLIP76 [6, 18, 19]. Hence, we performed drug-transport studies to see the effect of 2HF on ^14^C-DOX transport. The ATP-dependent ^14^C-DOX transport was conducted in purified reconstituted proteoliposomes prepared from MCF7 and MDA-MB-231 cells. The ^14^C-DOX transport was significantly inhibited by 2HF (10 and 20 μM) and anti-RLIP76 IgG (10 μg/ml). The combination of both anti-RLIP76 IgG (10 μg/ml) and 2HF (20 μM) resulted in stronger inhibition of ATP-dependent ^14^C-DOX transport activity (Figure 1C and 1D).

### Molecular docking studies on 2HF interaction with RLIP76, ERα and epidermal growth factor receptor 2 (HER2)

**Figure 2:**
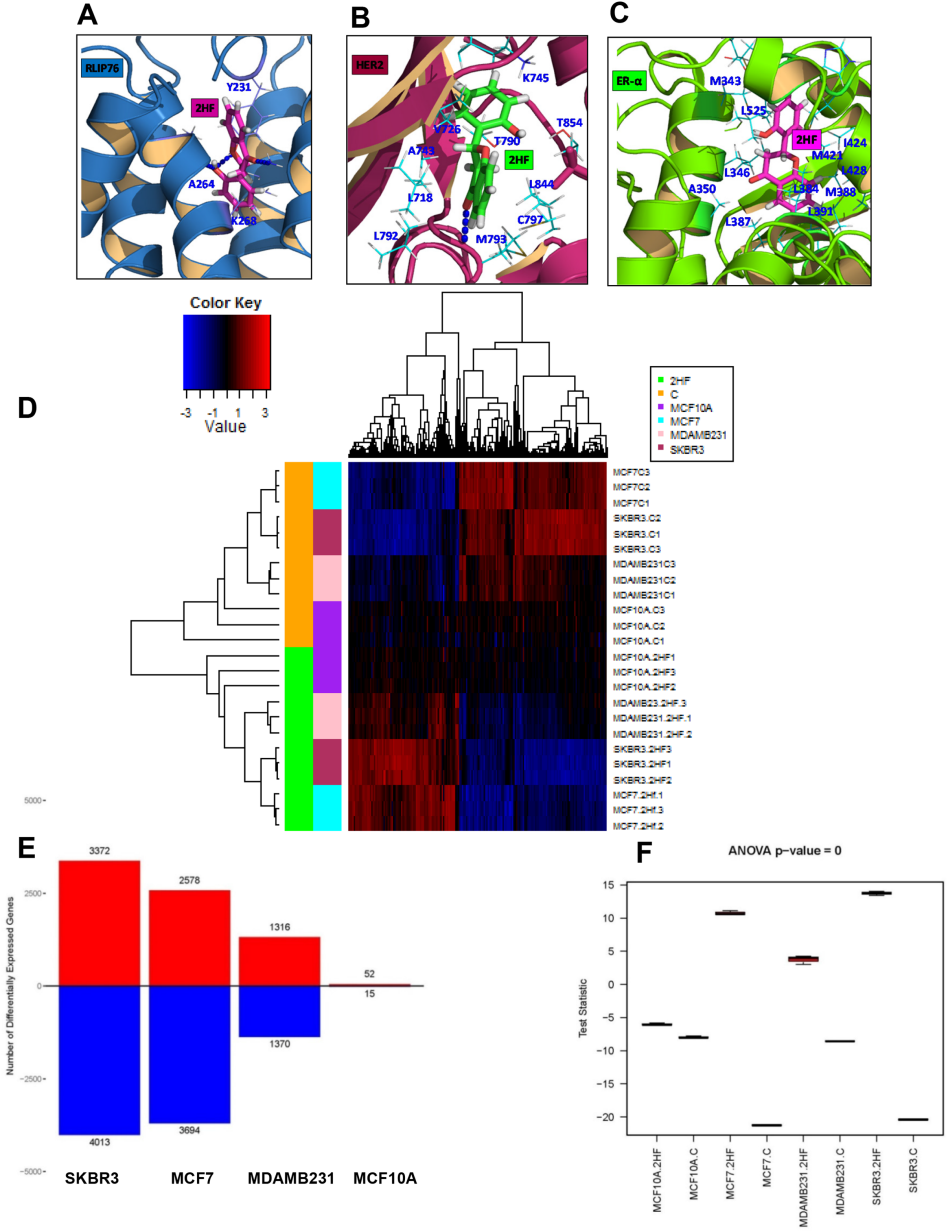
Docking of 2HF to RLIP76, ERα and HER2 and Effect on global gene expression in breast cancer cells The high Glide XP docking scores of 2HF implies that 2HF has high binding energy in complex with the proteins RLIP76, HER2 and ESR1. **(A)** 2HF forms a hydrogen bond network with the ligand (DNP-SG)-binding site of RLIP76 *via* Y^231^, K^268^ and A^264^ residues at a docking score of −10.5 kcal/mol. **(B)** 2HF also prefers binding at the ATP-binding pocket of HER2 forming hydrogen bond to M^793^ at a docking score of −10.2 kcal/mol. **(C)** 2HF docks to ERα at the ligand-binding site through hydrophobic interactions at a docking score of −9.7 kcal/mol. **(D)** Heatmap showing a major reversal of gene expression pattern in MCF7, MDA-MB-231 and SKBR3 cells with minimal impact on MCF10A normal breast epithelial cells. **(E)** Bar diagram showing number of differentially expressed genes in normal breast epithelial and BC cells. **(F)** Box-plot diagram showing the statistical significance of 2HF induced differential gene expression in normal breast epithelial and BC cells.

We conducted docking studies to study the interaction between 2HF and RLIP76, ERα and HER2 by employing Schordinger Glide docking software [20, 21]. 2HF formed hydrogen bonds with tyrosine (Y^231^), lysine (K^268^) and alanine (A^264^) residues of RLIP76 with a docking score of −10.5 kcal/mol at its previously known ligand (DNP-SG)-binding site [7, 10] (Figure 2A). 2HF also showed preferred binding at the ATP-binding pocket of HER2 by forming hydrogen bond to methionine (M^793^) with a docking score of −10.2 kcal/mol (Figure 2B). 2HF interacted with the ligand-binding site of ERα through hydrophobic interactions at a docking score of −9.7 kcal/mol (Figure 2C). These studies were informative on the potential of 2HF to act upon critical nodes of BC signaling by direct binding to RLIP76, ERα and HER2.

The docking ability of 2HF to RLIP76, ERα and HER2 along with its ability to enhance inhibitory effect of RLIP76 depletion on BC survival, and decrease RLIP76-mediated DOX transport together provided a strong rationale for the mechanistic regulatory role of 2HF in BC. In this regard, we further conducted gene expression studies following 2HF treatment in BC cells.

### Analyses of global gene expression changes following 2HF treatment

The MCF10A normal breast epithelial cells and ER^+^ MCF7, HER2^+^ SKBR3 and triple-negative MDA-MB-231 BC cells were treated with 50 μM 2HF, RNA was isolated at 24 h and RNA-Seq analyses was done at City of Hope Genomics Core as described in the *Methods Section*. Three biological replicates were used for each cell line and for each of the treatment groups. Reads were aligned using TopHat (2.0.8b) to human genome hg19 (Supplementary Tables 1 and 2). The gene expression profiles and curves (log2FPKM+0.1) of cell lines and treatment groups following 2HF treatment are presented in Supplementary Figure 1A and 1B. Read alignment statistics are provided in Supplementary Table 2. We further analyzed the statistically significant differences in gene expression (fold change ≤ 0.5 and ≥ 1.5 with *p* ≤ 0.05) using Ingenuity Pathway Analysis (Ingenuity systems, CA). The differential gene expression heatmap of 2HF treated and control groups showed a strong reversal of gene expression pattern in MCF7, MDA-MB-231 and SKBR3 BC cells while minimal impact was observed on the gene expression pattern of MCF10A normal breast epithelial cells (Figure 2D). The 2HF treatment led to statistically significant up regulation of 3372 genes and down regulation of 4013 genes in SKBR3 cells, up regulation of 2578 genes and down regulation of 3694 genes in MCF7 cells, and up regulation of 1316 genes and down regulation of 1370 genes in MDA-MB-231 cells. On the other hand, 2HF had modest effect in MCF10A cells as evident by up regulation of 52 genes and down regulation of 15 genes (Figure 2E). The Venn diagram showing the overlapping up regulated and down regulated genes is presented in Supplementary Figure 1C and 1D, respectively. The complete lists of differentially expressed genes following 2HF treatment in MCF10A, MCF7, MDA-MB-231 and SKBR3 cells are provided in Supplementary Tables 3-6. The box plot showing the statistical significance of 2HF induced differential gene expression is presented in Figure 2F.

### Regulation of critical survival, metastatic and differentiation genes by 2HF in breast cancer cells

The 2HF treatment led to significant changes in the expression of a number of genes with critical functional role in the incidence, progression and therapy-resistance of BC (Supplementary Table 7). AFAP1L2/XB130 is an adaptor protein that is associated with multiple tyrosine kinase signaling transduced through the PI3K/Akt pathway and regulates cell invasion and motility [22]. 2HF treatment led to strong down-regulation of AFAP1L2 in ER^+^ MCF7 cells. Carbonic anhydrase 9 (CA9) is involved in hypoxic survival of tumor cells and mediates resistance to both adjuvant chemotherapy and endocrine therapy in BC [23]. 2HF treatment led to a greater decrease in the levels of CA9 expression in MDA-MB-231 triple negative BC cells as compared to MCF7 and SKBR3 cells (Supplementary Table 7). The chloride intracellular channel protein 3 (CLIC3) is associated with tumor progression through its glutathione-dependent oxidoreductase activity [24]. The CLIC3 expression is associated with poor prognosis in early BC [25]. 2HF down-regulated the expression of CLIC3 with a predominant effect seen in ER^+^ MCF7 and HER2^+^ SKBR3 cells as compared to triple-negative MDA-MB-231 cells. The GABA type a receptor associated protein like 1 (GABARAPL1) is known to function as a tumor suppressor in BC by maintaining normal cellular metabolism [26]. 2HF treatment led to a strong up regulation of GABARAPL1 as in MCF7 and SKBR3 cells as compared to MDA-MB-231 cells. 2HF treatment also led to down-regulation of BC promoting miR210-HG in MCF7, MDA-MB-231 and SKBR3 cells [27].

We next assessed whether 2HF affects ERα and HER2 protein levels. 2HF decreased both ERα and HER2 protein levels in a concentration dependent manner in ER^+^ MCF7 and HER2^+^ SKBR3 BC cells, respectively (Supplementary Figure 2A and 2B). An ongoing clinical trial (NCT00338728) is investigating the combination of letrozole, an aromatase inhibitor which decreases the levels of estrogen, and imatinib mesylate in hormone-sensitive and advanced BC. As 2HF directly binds to ERα, we further assessed the efficacy of 2HF on cell survival in combination with imatinib mesylate (Gleevec). The 25 and 50 μM 2HF treatment significantly enhanced efficacy of 5 and 10 μM imatinib mesylate in ER^+^ MCF7 cells, a finding which indicated the ability of 2HF to sensitize BC cells to imatinib mesylate therapy (Supplementary Figure 2C). We further confirmed 2HF induced gene expression changes by qRT-PCR (Supplementary Figure 2D-2F). The miR-22, a tumor suppressor miRNA and JMY, a P300 dependent activator of p53, were significantly increased in MCF7, MDA-MB-231 and SKBR3 cells [28, 29]. MiR-22 is also known to inhibit the function of ERα in BC [29]. ITGB6 is a prognostic marker associated with breast tumor progression [30]. We observed a down-regulation of ITGB6 in 2HF treated HER2^+^ SKBR3 and triple-negative MDA-MB-231 cells (Supplementary Figure 2E and 2F).

### Ingenuity pathway analyses (IPA) of differentially regulated gene networks

We further conducted IPA analyses to assess the significance of 2HF induced differential gene expression in regulating cellular signaling networks. The Supplementary Tables 8-11 provide information on all the upstream molecules differentially regulated following 2HF treatment in MCF10A normal breast epithelial and MCF7, MDA-MB-231 and SKBR3 BC cells. The differentially regulated top canonical pathways are presented in Supplementary Figure 3-6.

**Figure 3:**
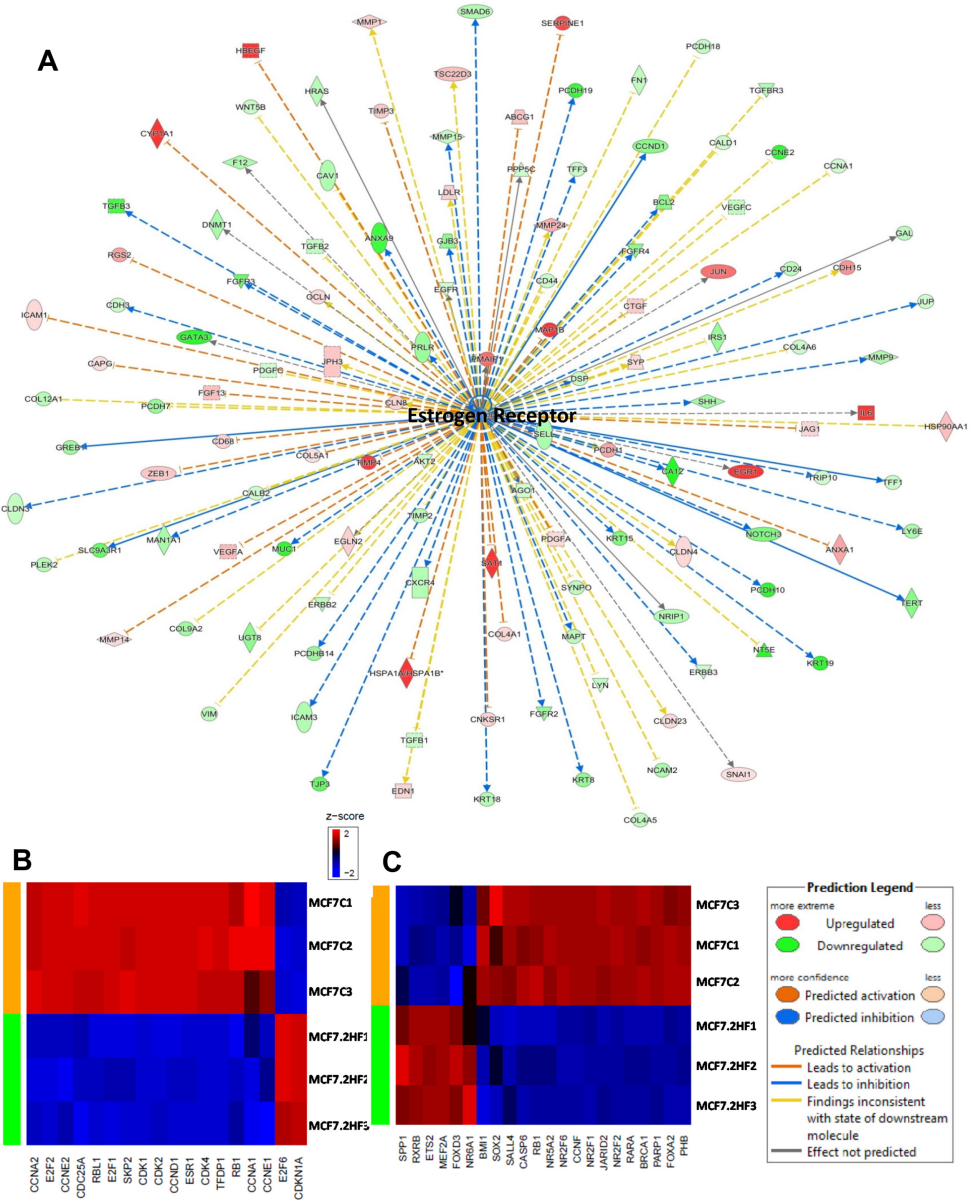
Effect of 2HF induced gene expression changes in ER+ MCF7 breast cancer cells **(A)** Ingenuity Pathway Analyses (IPA) revealing the differentially expressed ER network genes in ER^+^ MCF7 cells. **(B)** Heatmap showing 2HF induced differential gene expression in “Estrogen mediated s-phase entry canonical pathway” in MCF7 cells. **(C)** Heatmap showing 2HF induced differential gene expression in “Role of Oct4 in Mammalian Embryonic Stem cell pluripotency pathway” in MCF7 cells.

2HF treatment led to inhibition of estrogen network in ER^+^ MCF7 cells (z score: - 2.363 and *p*: 5.4×10^−5^) (Figure 3A). Expression of target genes like TGFβ1, BCL2, and TERT, which are up-regulated by estrogen-receptor, was decreased while expression of genes COL4A1 and RGS2, which are down-regulated by estrogen-receptor, was increased following 2HF treatment in MCF7 cells (Figure 3A). In addition, 2HF treatment led to inhibition of estrogen-mediated S-phase entry canonical pathway in MCF7 cells (Figure 3B). Along with ESR1/ERα network, estrogen-mediated canonical pathway genes like CCNA2, CCNE2, E2F1, CDK1, CDK2 and CDK4 were down regulated in MCF7 cells (Figure 3B, *p*: 9.65×10^−4^). E2F6 is known to be a dominant negative inhibitor of the E2F family of transcription factors while CDKN1A/p21 is a p53 induced negative inhibitor of cell cycle [31, 32]. 2HF treatment led to up regulation of E2F6 and CDKN1A (Figure 3B). In addition, *Oct4* canonical pathway was strongly inhibited along with down-regulation of stem cell pluripotency regulatory genes including SOX2, JARID2, and PHB following 2HF treatment in ER^+^ MCF7 cells (Figure 3C, *p*: 0.02). The ATP-P2RY2- β-catenin pathway is known to promote invasion and metastases [33]. 2HF treatment down-regulated the P2RY2 purinergic receptor signaling pathway in MCF7 cells (Supplementary Figure 7). Sphingosine-1-phosphate is elevated in BC and plays a major role *via* phospholipase C (PLC) and RAS induced invasion [34]. The sphingosine-1-phosphate network was down-regulated by 2HF treatment in ER^+^ MCF7 cells (Supplementary Table 7, Supplementary Figure 8).

**Figure 4:**
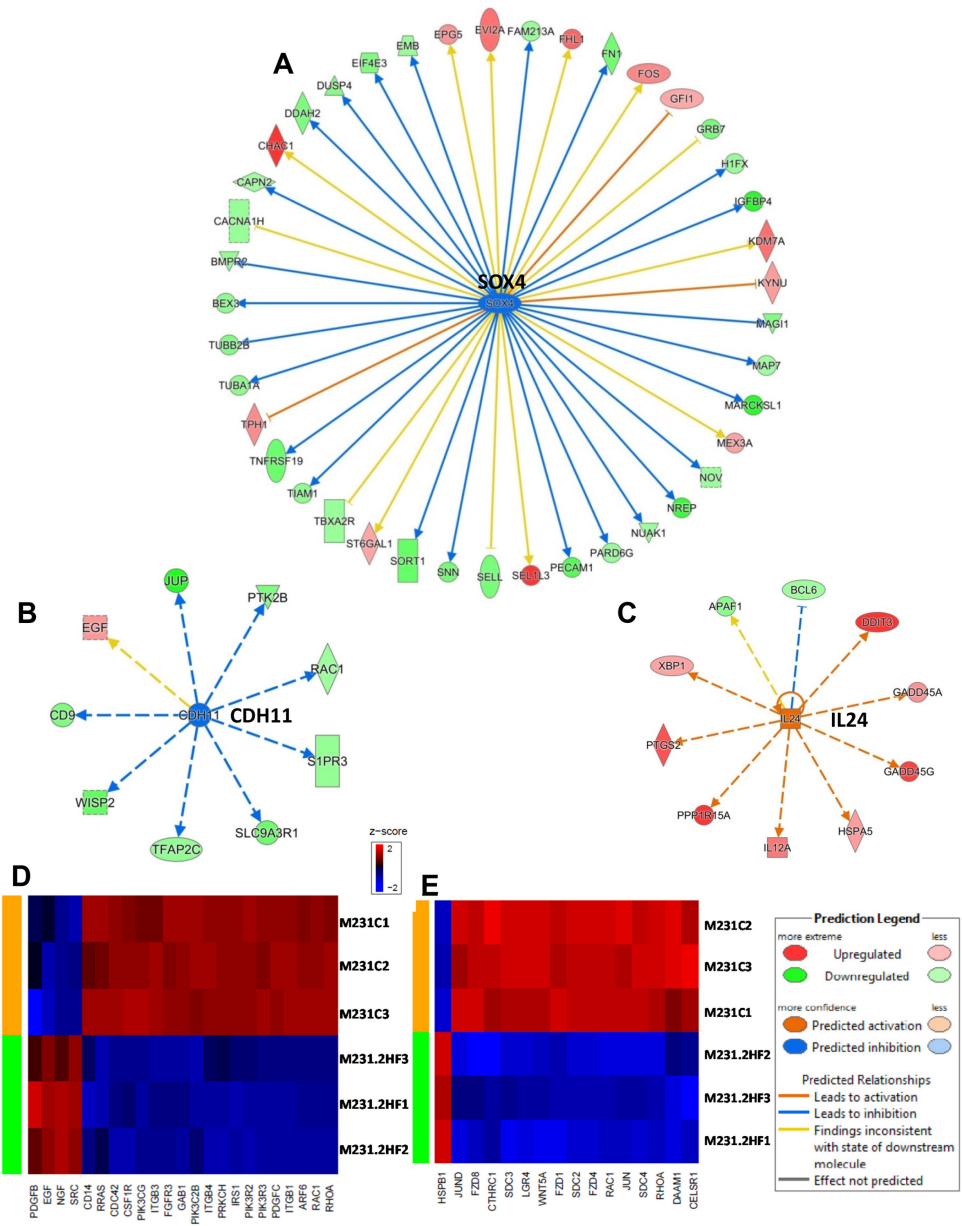
Effect of 2HF induced gene expression changes in triple-negative MDA-MB-231 breast cancer cells **(A)** Ingenuity Pathway Analyses (IPA) revealing the differentially expressed SOX4 network genes in triple-negative MDA-MB-231 cells. **(B)** IPA revealing the differentially expressed CDH11 network genes in MDA-MB-231 cells. **(C)** IPA revealing the differentially expressed IL24 network genes in MDA-MB-231 cells. **(D)** Heatmap showing 2HF induced differential gene expression in “Macropinocytosis signaling canonical pathway” in MDA-MB-231 cells. **(E)** Heatmap showing 2HF induced differential gene expression in “Planar cell polarity (PCP) pathway” in MDA-MB-231 cells.

SOX4 promotes PI3K/Akt signaling along with mediating TGFβ induced epithelial-mesenchymal transition (EMT) in BC cells [35]. The expression of CDH11 is increased in early stage and basal like BCs and mediates stem cell fate decisions [36]. 2HF treatment down-regulated SOX4 and CDH11 networks in MDA-MB-231 cells (Figure 4A, z score: - 2.2 and *p*: 7.19×10^−5^) and Figure 4B, z score: −2.33 and *p*: 0.01). The expression of IL24 tumor suppressor is lost in basal like BC [37]. 2HF treatment activated the IL24 network functions in basal like triple-negative MDA-MB-231 BC cells (Figure 4C, z score: 2.76 and *p*: 0.02). Macropinocytosis is an actin driven endocytic process that is known to regulate EGF, PDGF and HGF signaling [38]. 2HF treatment inhibited the macropinocytosis pathway in triple-negative MDA-MB-231 cells with a marked decrease in the expression of macropinocytosis associated genes including RRAS, RAC1, CDC42, and ARF6 (Figure 4D, *p*: 0.004). The planar cell polarity (PCP) pathway involving WNTs and frizzled (FZD) receptors controls the distribution of cells within a plane that in turn influences the invasiveness and migration of BC cells [39]. 2HF treatment led to down-regulation of PCP pathway (Figure 4E, *p*: 0.016). The down-regulation of PCP pathway in MDA-MB-231 cells was characterized by decreased expression of FZD1, FZD4 and FZD8 receptors, LGR4, syndecans 3 and 4 (SDC3 and SDC4), which are associated with WNT signaling (Figure 4E & Supplementary Figure 9). Further, 2HF treatment induced a down-regulation of multiple tubulin isoforms 1, 2, 3 and 4 *via* down-regulation of 14-3-3 signaling while also inhibiting IGF1 signaling pathway, which is activated in triple-negative BCs (Supplementary Figures 10 and 11) [40, 41].

**Figure 5:**
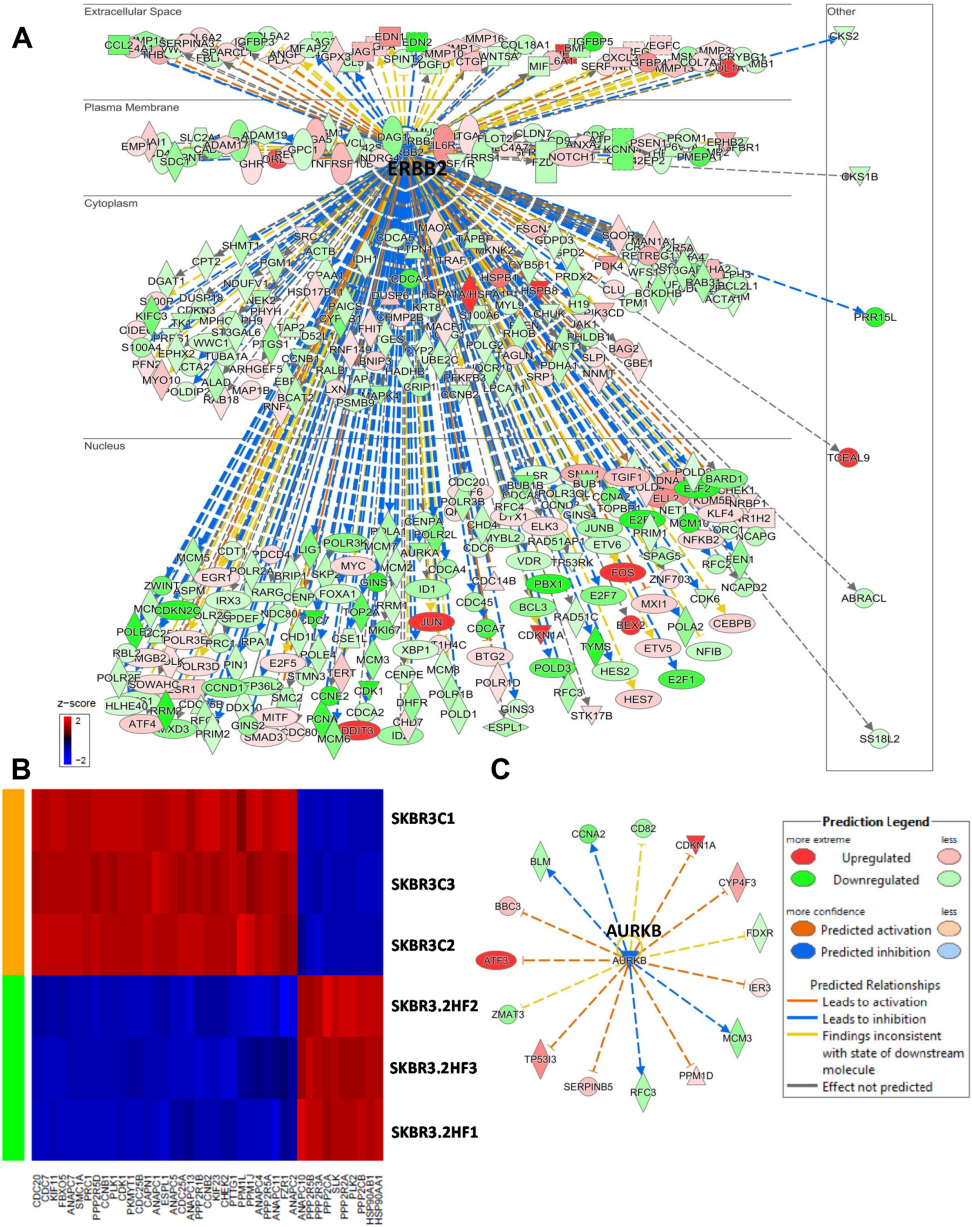
Effect of 2HF induced gene expression changes in HER2+ SKBR3 breast cancer cells **(A)** Ingenuity Pathway Analyses (IPA) revealing the differentially expressed HER2 network genes in HER2^+^ SKBR3 cells. **(B)** Heatmap showing 2HF induced differential gene expression in “Mitotic Role of Polo-Like kinase pathway” in SKBR3 cells. **(C)** IPA revealing the differentially expressed AURKB network genes in SKBR3 cells.

In HER2^+^ SKBR3 cells, 2HF treatment led to decreased expression of HER2 and down-regulation of HER2 signaling network (Figure 5A, z score: - 4.798 and *p*: 2.34×10^−8^). Polo-Like kinases (PLK) are serine/threonine kinases that regulate multiple functions during cell cycle [42]. 2HF treatment led to inhibition of polo-like kinase mediated mitotic activity pathway in HER2^+^ SKBR3 cells (Figure 5B & Supplementary Figure 12, *p*: 5.88×10^−4^). The AURKB expression is correlated with strong proliferative index and chemo-resistance in BC [43]. 2HF inhibited AURKB network in HER2^+^ SKBR3 cells (Figure 5C, *p*: 0.007). Sphingosine-1-phosphate signaling mediates phospholipase C (PLC) and RAS induced invasion [34]. 2HF treatment also led to inhibition of Sphingosine-1-phosphate signaling in HER2^+^ SKBR3 cells (Supplementary Figure 13).

### 2HF treatment regulates critical genes of clinical-prognostic significance

MammaPrint is a FDA approved test containing a panel of genes to aid therapeutic decisions in women of all ages with both ER^+^ and ER^−^ BCs. List of differentially regulated MammaPrint genes following 2HF treatment in MCF7, MDA-MB-231 and SKBR3 cells are presented in Supplementary Table 12. 2HF treatment led to down-regulation of MammaPrint genes CCNE2, MCM6, DCK, LPCAT1, DTL, GNAZ, ECI2, MELK, NMU, NUSAP1 and EGLN1 in BC cells. Transforming Growth Factor beta 3 (TGFβ3) is expressed at high levels in primary BC [44]. TGFβ3 is known to act as a tumor promoter in triple-negative BCs [45]. Both, TGFβ3 and TGFβ receptor 3 (TGFβR3) were down-regulated in 2HF treated MCF7 cells. While TGFβ3 changes were undetected, TGFβR3 was down-regulated by 5 folds in MDA-MB-231cell lines (Supplementary Table 5). Thus, down-regulation of TGFβ3 pathway was common to both 2HF treated ER^+^ MCF7 and triple-negative MDA-MB-231 cells.

**Figure 6:**
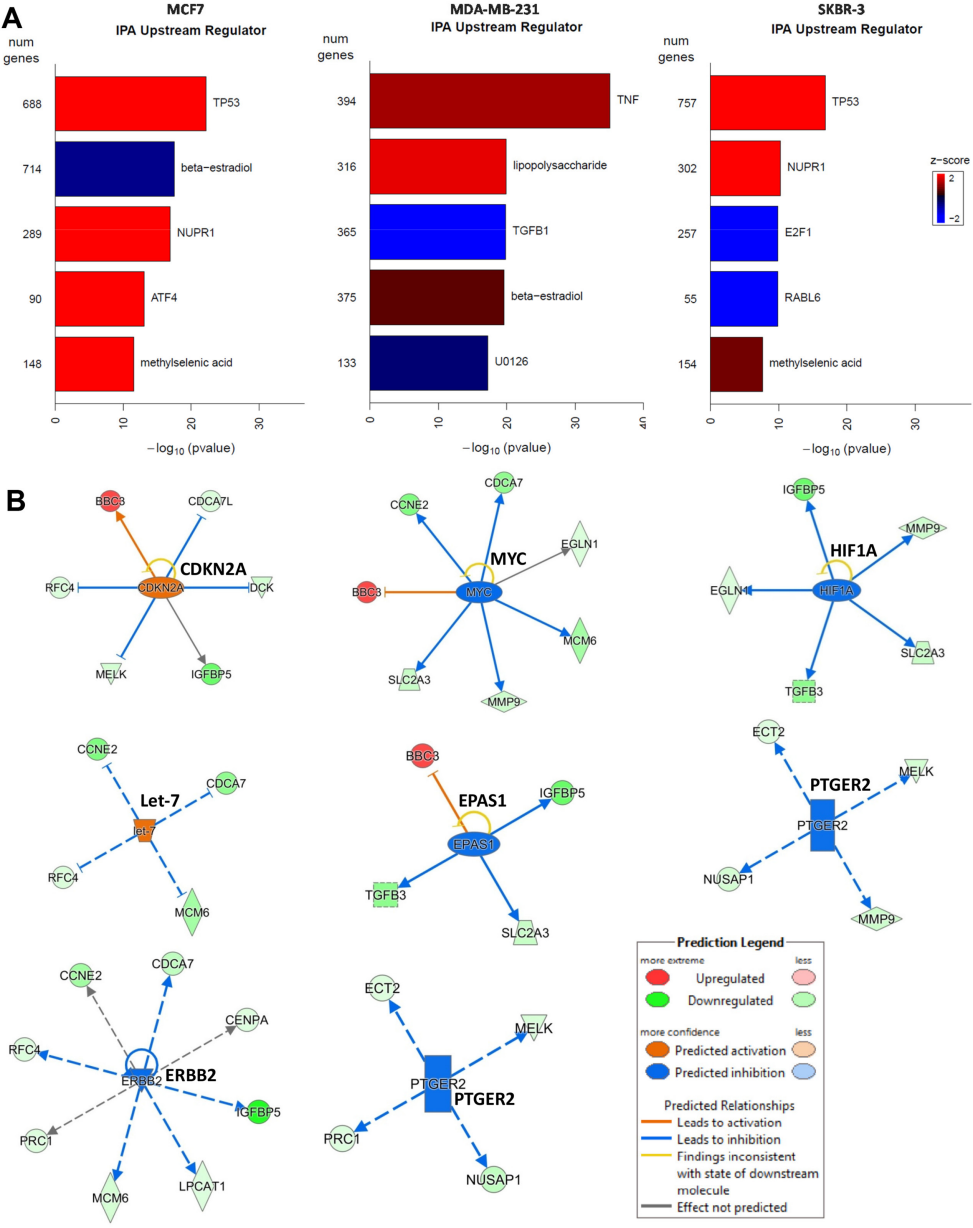
Effect of 2HF on critical upstream regulators in breast cancer cells **(A)** Ingenuity Pathway Analyses (IPA) revealing the differential regulation of key upstream targets including TP53, estradiol, TGFβ1, E2F1, RABL6 in BC cells. **(B)** 2HF induced regulation of critical nodes like CDKN2A, MYC, HIF1A, let-7, EPAS1, PTGER2 and ERBB2 that in turn regulate a number of MammaPrint clinical-prognostic gene panel.

The top upstream changes that were differentially regulated following 2HF treatment were TP53 activation and β-estradiol inhibition in ER^+^ MCF7 cells, TGFβ1 inhibition in triple-negative MDA-MB-231 cells, and TP53 activation, E2F1 and RABL6 inhibition in HER2^+^ SKBR3 cells (Figure 6A). The regulation of MammaPrint genes has been predicted to be due to 5 upstream nodes including TP53, RB1, MYC, JUN and CDKN2A [46]. In this regard, using IPA, we further analyzed the differences in 2HF induced fold changes of MammaPrint genes for each of the respective cell lines (Figure 6B). We did not observe a particular pattern of MammaPrint upstream nodes in triple-negative MDA-MB-231 cells. IPA revealed that the 2HF induced MammaPrint gene expression is regulated by down-regulation of EPAS1, MYC, HIF1A and PTGER2 and by up regulation of CDKN2A and let-7 networks in ER^+^ MCF7 cells. Down-regulation of ERBB2/HER2 and PTGER2, and up regulation of let-7 networks contributed to many of the changes in 2HF induced expression of MammaPrint genes in HER2^+^ SKBR3 cells. These findings, given the known ability of 2HF to decrease RLIP76 levels, when taken in the context of the role of RLIP76 in receptor-ligand signaling mediated by CDE and in pathogenesis of metabolic syndrome, provide promising evidence for 2HF and RLIP76 focused interventions for targeting both BC risk and progression [15, 47–48].

## DISCUSSION

Characterization of novel therapeutic molecules capable of targeting critical mechanistic nodes in BC is a significant focus of translational cancer research. RLIP76 is a multi-functional protein: a major and multi-specific GS-E transporter of MAP that regulates apoptotic resistance *via* mediating efflux of products of lipid peroxidation and facilitates tumor drug-resistance by mediating the transport of GS-Es of chemotherapy drugs [6–8]. RLIP76 is a rac and ral effector and has been shown to be an essential factor in determining the invasive and metastatic aggressiveness of cancers [49–51]. Importantly, RLIP76 has been shown to be an essential prerequisite for epithelial carcinogenesis [7]. The expression of RLIP76 is up-regulated in multiple cancers while inhibiting either the function or expression of RLIP76 causes regression of established xenografts of skin, lung, pancreatic, kidney and colon cancers [8–10]. Our studies indicate that RLIP76 inhibition in combination with 2HF could be directly relevant to therapeutic management of BC. RLIP76 inhibition along with 2HF administration is also a translationally significant chemoprevention strategy that needs to be further considered as RLIP76 knockout mice survive full life-span without any overt toxicity.

Multiple findings from this study provide a highly significant and directly relevant mechanistic evidence for further development and clinical testing of 2HF in BC. First, 2HF shows docking to ligand (DNP-SG)-binding site of RLIP76, ATP binding site of HER2 and ligand-binding site of ERα. Second, 2HF decreases the levels of ERα, HER2 and potentiates the effects of RLIP76 antibody in both inhibiting BC cell survival and in doxorubicin transport studies. Third, 2HF induces strong regulatory effects at gene expression levels in all the major types of BC cells. In support of our docking model, the gene expression studies confirmed a comprehensive down-regulation of ERα and HER2 networks. Fourth, 2HF was effective in triple-negative MDA-MB-231 cells where it induced significant inhibition of macropinocytosis and Planar Cell Polarity Pathway (PCP) signaling, a significant finding given the established role of RLIP76 in regulating endocytosis of ligand-receptor complexes and Wnt-β catenin signaling. Fifth, the translational relevance of 2HF was further confirmed by its characteristic down-regulation of critical MammaPrint genes including CCNE2, LPCAT1, MCM6, DCK, GNAZ, ECI2, DTL, MELK, NUSAP, NMU, 1and EGLN1 in MCF7, SKBR3 and MDA-MB-231 BC cells. In addition, 2HF regulated critical upstream regulators of MammaPrint genes including TP53, MYC and CDKN2A. Given the positive association of obesity with BC incidence and progression in post-menopausal women and the known ability of flavonoids to decrease pro-inflammatory state induced by obesity, 2HF administration may also provide further translationally relevant benefits for BC risk reduction [52]. These factors provide collective basis for the strong chemopreventive and therapeutic potential of 2HF in BC.

In conclusion, the mechanistic spectrum of the effects of 2HF on RLIP76-mediated DOX transport, ERα, HER2, and MammaPrint prognostic gene networks collectively provides a strong rationale for translational development and clinical testing of 2HF and RLIP76 based interventions for BC prevention and therapy.

## MATERIALS AND METHODS

### Reagents and cell lines

2HF (purity ~99%), Horseradish peroxidase (HRP)-conjugated anti-mouse, and anti-rabbit secondary antibodies, and MTT were purchased from Sigma-Aldrich, St. Louis, MO. The primary antibodies were purchased from Thermo Fisher, Abcam and Cell Signaling Technologies. ^14^C-DOX (specific activity 58 mCi/mmol) was purchased from NEN Life Sciences (Boston, MA). Human breast untransformed (MCF10A) and cancer (MCF7, MDA-MB-231 and SKBR3) cell lines were purchased from the American Type Culture Collection (ATCC, Manassas, VA). The authentication of cell lines was done by analyzing fifteen different human short tandem repeat (STR) at Genomic Core of the City of Hope, Duarte, CA. All the cells were also tested for Mycoplasma once every 3 months.

### Ethics statement

No animals and human subjects are involved in the present study.

### Drug sensitivity (MTT) assay

Cell density measurements were performed using a hemocytometer to count viable cells resistant to staining with trypan blue. Approximately 10,000 cells were plated into each well of 96-well flat-bottomed micro-titer plates. After 12 h incubation at 37 °C, medium containing 2HF (ranging 0-100 μM) were added to the cells. After 48 h incubation, 20 μl of 5 mg/ml MTT were introduced to each well and incubated for 2 h. The plates were centrifuged and medium was decanted. Cells were subsequently dissolved in 100 μl DMSO with gentle shaking for 2 h at room temperature, followed by measurement of OD_570_. Eight replicate wells were used at each point in each of three separate measurements.

### Computational and docking studies

Computational analyses of Lipinski Rule of 5 were conducted using TargetNet (http://targetnet.scbdd.com/calcnet/index_rule/). The binding models of 2HF molecule in complex with RLIP76, HER2 and ERα were calculated by using Schordinger induce fit docking software and our in-house developed All-Around Docking (AAD) methodology [20, 21]. The three-dimensional protein structures were obtained from protein data bank (PDB) as PDB id 2MBG for RLIP76, 1XKK for HER2 and 3ERT for ERα. AAD docking, which docks the small molecule on the whole surface of target protein to determine the best binding region, was carried out first to predict the binding pocket of 2HF. Then induced fit docking, which allows side-chain of the pocket residues to be flexible for more accurate calculation, was used to determine the docking score by docking 2HF to the binding pocket predicted from AAD.

### Purification and reconstitution of purified RLIP76 into artificial liposomes

DNPSG-affinity purification of RLIP76 from MCF7 and MDA-MB231 cells was carried out as described previously [18]. Purifications were monitored by measuring ATPase and transport activity. Purified RLIP76 from BC cells was dialyzed against reconstitution buffer (10 mM Tris-HCl, pH 7.4, 4 mM MgCl_2_, 1 mM EGTA, 100 mM KCl, 40 mM sucrose, 2.8 mM β-mercaptoethanol, 0.05 mM BHT, and 0.025% polidocanol), and reconstituted into artificial asolectin-cholesterol liposomes. An aqueous emulsion of soybean asolectin (40 mg/ml) and cholesterol (10 mg/ml) was prepared in the reconstitution buffer by sonication, from which a 100 μl aliquot was added to 0.9 ml of dialyzed purified RLIP76 protein. After sonication of the resulting mixture for 30 s at 50 W, 200 mg of SM-2 Bio-beads pre-equilibrated with reconstitution buffer (without polidocanol) were added to initiate vesiculation, and after 4 h incubation at 4 °C, SM-2 beads were removed by centrifugation at 3000 x g and the vesicles (proteoliposomes) were collected. Control-liposomes were prepared using an equal amount of crude protein from *E. coli* not expressing RLIP76. The size of reconstituted vesicles was examined by electron microscopy and intra-vesicular volume was estimated by ^14^C-inulin trapping [19].

### Transport studies in RLIP76-proteoliposomes

Transport studies of ^14^C-DOX in reconstituted vesicles were performed by rapid-filtration technique as described by us using 250 ng protein per 30 μl reaction mixture. ATP-dependent uptake of ^14^C-DOX (specific activity 8.5 × 10^4^ cpm/nmol, use 3.6 μM final concentration) was determined by subtracting the radioactivity (cpm) of the control without ATP from that of the experimental containing ATP, and the transport of DOX was calculated in terms of nmol/min/mg protein. Liposomes prepared without addition of RLIP76 were used for controls. Each determination was performed in triplicate [6].

### Transport inhibition by 2HF and anti-RLIP76 IgG

Purified reconstituted liposomes (250 ng protein/30 μl reaction mixture) were incubated separately with either 2HF (0-50 μM final concentration) or anti-RLIP76 IgG (0-60 μg/ml final concentration) or both, for 30 min at room temperature. In one of the controls, IgG was excluded while the other control was treated with an equal amount of pre-immune IgG. After incubation, the ATP-dependent transport of ^14^C-DOX was measured by using a 96 well-plate filtration manifold to separate the extra-vesicular drug from that taken up by the vesicles. Uptake was measured in parallel in RLIP76-proteoliposomes and control liposomes, in absence or presence of 2HF, anti-RLIP76 IgG and 4 mM ATP at a fixed time point of 5 min, at 37 °C [6].

### RNA isolation and sequencing

The cells (3 × 10^5^/well in 6-well plates) were treated with 50 μM 2HF or vehicle (DMSO) and RNA isolated 24 h post treatment. Three biological replicates were prepared for 2HF treatment as well as control untreated cells. After treatment, total RNA was prepared using RNeasy Mini Kit (Qiagen) according to the manufacturer's instructions, eluted in 50 μL of RNase/DNase-free water, and initial concentration and purity assessed by NanoDrop ND-1000 spectrophotometer (NanoDrop Technologies, Wilmington, DE). Prior to sequencing, RNA quality was also assessed by microfluidic capillary electrophoresis using an Agilent 2100 Bioanalyzer and the RNA 6000 Nano Chip kit (Agilent Technologies, Santa Clara, CA). Sequencing libraries were prepared with the TruSeq RNA Sample Prep Kit V2 (Illumina, San Diego, CA) according to manufacturer's protocol with minor modifications. Briefly, ribosomal RNA was removed from 500 ng of total RNA using RiboZero kit (Illumina) and the resulting RNA was ethanol precipitated. Pellets were resuspended in 17 μl of Elute/Prime/Fragment Mix (Illumina) and first-strand cDNA synthesis performed using DNA polymerase I and RNase H. cDNA was end repaired, 3’ end adenylated, and universal adapter ligated followed by 10 cycles of PCR using Illumina PCR Primer Cocktail and Phusion DNA polymerase (Illumina). Libraries were purified with Agencourt AMPure XP beads, validated with Agilent Bioanalyzer 2100, and quantified with Qubit (Life Technologies). Libraries were sequenced on Illumina Hiseq 2500 with single end 40 bp reads. Reads were aligned using TopHat (2.0.8b) to human genome hg19. Read alignment statistics are provided in Supplementary Tables 1 and 2.

### RNA-seq and gene ontology

Differential gene expression was identified from standard Partek workflow (Partek Genomics Suite v6.6, Partek, Inc) using ANOVA, with step-up FDR multiple testing correction *p*-value <0.05 and requiring a >1.5x fold change between treatment and control samples. Gene ontology for the 24 h 2HF treatment up- or down-regulated genes were analyzed for functional enrichment using the Database for Annotation, Visualization, and Integrated Discovery (DAVID, v6.7). For inclusion, terms required an EASE score of *p*<0.005. Fold-changes in gene expression between control and 24 h 2HF treatment were derived from the comparative *CT* method with β-actin as an internal control. Correlation between the expression values detected by RNA-seq (normalized log2 RPKM fold-change) and qRT-PCR (mean fold change) for the 10 genes tested was estimated by calculating Spearman's Rho correlation in the Prism 6.0 software (GraphPad, San Diego, CA, USA). Mann-Whitney U-tests were used to assess statistical significance between time points (two-tailed,^*^ *p*<0.05). The significance of differentially regulated genes was further analyzed by Ingenuity Pathway Analyses (IPA; Ingenuity Systems, CA). The statistically significant gene expression fold differences from 2HF and control samples for each of the cell lines were thoroughly analyzed using IPA. The differences in canonical pathways, upstream regulators and networks were analyzed using IPA along with further search for latest findings on key nodes of signaling regulators as relevant to pathogenesis and clinical prognosis of breast cancer.

### Quantitative real-time PCR (qRT-PCR) validation

To confirm the results obtained from RNA-seq analysis, biologically significant up- and down-regulated genes at 24 h post 2HF treatment were selected and analyzed by qRT-PCR. First strand cDNA was prepared using the High Capacity cDNA Reverse Transcription Kit (Life Technologies). The primer pairs were purchased from Integrated DNA Technologies (San Diego, CA). The qRT-PCR was performed on three independent samples per treatment using the ABI-7500 Fast Real Time PCR system (Life Technologies) and Power SYBR Green master mix. After initial incubation for 2 min at 50 °C, the cDNA was denatured at 95 °C for 10 min followed by 40 cycles of PCR (95 °C for 15 s, 60 °C for 60 s).

### Immunoblotting

Supernatant proteins from control and 2HF treated cell lysates were resolved by sodium-dodecyl sulfate polyacrylamide gel electrophoresis and transferred onto polyvinylidene fluoride membrane. Change in the level of desired protein was determined by densitometric scanning of the immuno-reactive bands. Equal loading of proteins was confirmed by stripping and re-probing the membranes with β-actin antibodies.

### Statistical analysis

All data were evaluated with a two-tailed unpaired student's *t* test are expressed as the mean ± SD. The statistical significance of differences between control and treatment groups was determined by ANOVA followed by multiple comparison tests. Differences were considered statistically significant when z score was either above 2 or below −2, and the *p* value was less than 0.05.

## SUPPLEMENTARY MATERIALS FIGURES AND TABLES





## Abbreviations

AKT: protein kinase B (PKB), also known as AKT
pAMPK: phosphorylated form of AMP-activated protein kinase
BC: breast cancer
BCL2: B-cell CLL/lymphoma 2
CDE: clathrin-dependent endocytosis
CDK: cyclin dependent kinase
E2F1: E2F transcription factor 1
EGF: epidermal growth factor
EMT: epithelial mesenchymal transition
ER: estrogen receptor
EGFR: epidermal growth factor receptor
ERBB2: Erb-B2 receptor tyrosine kinase 2 or HER2
ERK: extracellular signal-regulated kinase
FDA: Food and Drug Administration
HBA: Hydrogen Bond Acceptors
HBD: Hydrogen Bond Donors
HER2: receptor tyrosine-protein kinase erbB-2
ITGA: integrinA
ITGB6: integrin beta 6
IGF: insulin growth factor
IPA: ingenuity pathway analyses
logP: Partition Coefficient
miR: micro-RNA
MMP: matrix metallo proteinase
MR: Molar Refractivity
PI3K: phosphoinositide-3 kinase
RLIP76: 76 kDa a ral binding/ral-interacting protein, also known as RalBP1
TP53: tumor suppressor 53
TPSA: total polar surface area
SOX4: SRY-Box 4
TGFβ3: transforming growth factor beta 3
