# Correction: 2’-Hydroxyflavanone effectively targets RLIP76-mediated drug transport and regulates critical signaling networks in breast cancer

**DOI:** 10.18632/oncotarget.28199

**Published:** 2022-02-11

**Authors:** Lokesh Dalasanur Nagaprashantha, Jyotsana Singhal, Hongzhi Li, Charles Warden, Xueli Liu, David Horne, Sanjay Awasthi, Ravi Salgia, Sharad S. Singhal

**Affiliations:** ^1^Department of Medical Oncology, Beckman Research Institute of City of Hope, Comprehensive Cancer Center and National Medical Center, Duarte, CA 91010, USA; ^2^Department of Molecular Medicine, Beckman Research Institute of City of Hope, Comprehensive Cancer Center and National Medical Center, Duarte, CA 91010, USA; ^3^Department of Computational Therapeutics, Beckman Research Institute of City of Hope, Comprehensive Cancer Center and National Medical Center, Duarte, CA 91010, USA; ^4^Department of Genomic Core, Beckman Research Institute of City of Hope, Comprehensive Cancer Center and National Medical Center, Duarte, CA 91010, USA; ^5^Department of Information Sciences & Biostatistics, Beckman Research Institute of City of Hope, Comprehensive Cancer Center and National Medical Center, Duarte, CA 91010, USA; ^6^Department of Internal Medicine, Texas Tech University Health Sciences Center, Lubbock, TX 79430, USA

**This article has been corrected:** A minor typo has been corrected in the *Analyses of global gene expression changes following 2HF treatment* paragraph. The 4th sentence should read, ‘The gene expression profiles and curves log2(FPKM+0.1) of cell lines and treatment groups following 2HF treatment are presented in Supplementary Figure 1A and 1B.’ In addition, in the Materials and Methods section, the *RNA-seq and gene ontology* paragraph has been updated. The new text is shown below.

Original article: Oncotarget. 2018; 9:18053–18068. 18053-18068. https://doi.org/10.18632/oncotarget.24720

Read counts were tabulated using htseq-count [1], with UCSC known gene annotations (TxDb.Hsapiens.UCSC. hg19.knownGene, [2]). Fold-change values were calculated from Fragments Per Kilobase per Million reads (FPKM, [3]) normalized expression values, which were also used for visualization (following a log 2 transformation). Aligned reads were counted using GenomicRanges [4]. Prior to p-value calculation, genes were filtered to only include transcripts with an FPKM expression level of 0.1 (after a rounded log2- transformation) in at least 50% of samples [5] as well as genes that are greater than 150 bp.

Differentially expressed genes were defined using two strategies. For the 1 st strategy, a 2-variable model was used to compare differences between 2HF and Control samples, adjusting for differences between cell lines. For the 2 nd strategy, 2HF versus Control comparisons were run for each cell line. In both cases, *p*-values were calculated from raw counts using DESeq2 [6], and false discovery rate (FDR) values were calculated using the method of Benjamini and Hochberg [7]; genes were defined as differentially expressed if they had a |fold-change| > 1.5 and FDR < 0.05. A heatmap to visualize differentially expressed genes from the 2-variable model (with two annotation columns) was created using the heatmap.3() function (https://github.com/obigriffith/biostar-tutorials/blob/master/Heatmaps/heatmap.3.R) in R. Heatmaps for differentially expressed genes within GO or IPA gene sets for individual cell lines (with one annotation column) were created using heatmap.2() in the ‘gplots’ package. Barplots were created using the barplot() function in R. Gene Ontology (GO [8] ) enrichment was calculated using goseq [9]. Additional systems-level analysis was performed in IPA (Ingenuity® Systems, https://www.ingenuity.com). The genes from the 2-variable DESeq2 differential expression comparison were used to define signature in BD-Func [10], which was then applied to produce scores on a per-sample basis (from log2(FPKM + 0.1) values), as an alternative strategy to view the difference in scores between 2HF and Control samples.

