# Supplementary material for: 2’-Hydroxyflavanone effectively targets RLIP76-mediated drug transport and regulates critical signaling networks in breast cancer
Source: Oncotarget. 2018 Apr 6;9(26):18053–68. doi: 10.18632/oncotarget.24720 (PMC5915057; doi:10.18632/oncotarget.24720)
Supplement: Supplementary file 1 [file oncotarget-09-18053-s001.pdf]

2'-Hydroxyflavanone effectively targets RLIP76-mediated drug transport and regulates critical signaling networks in breast cancer

SUPPLEMENTARY MATERIALS

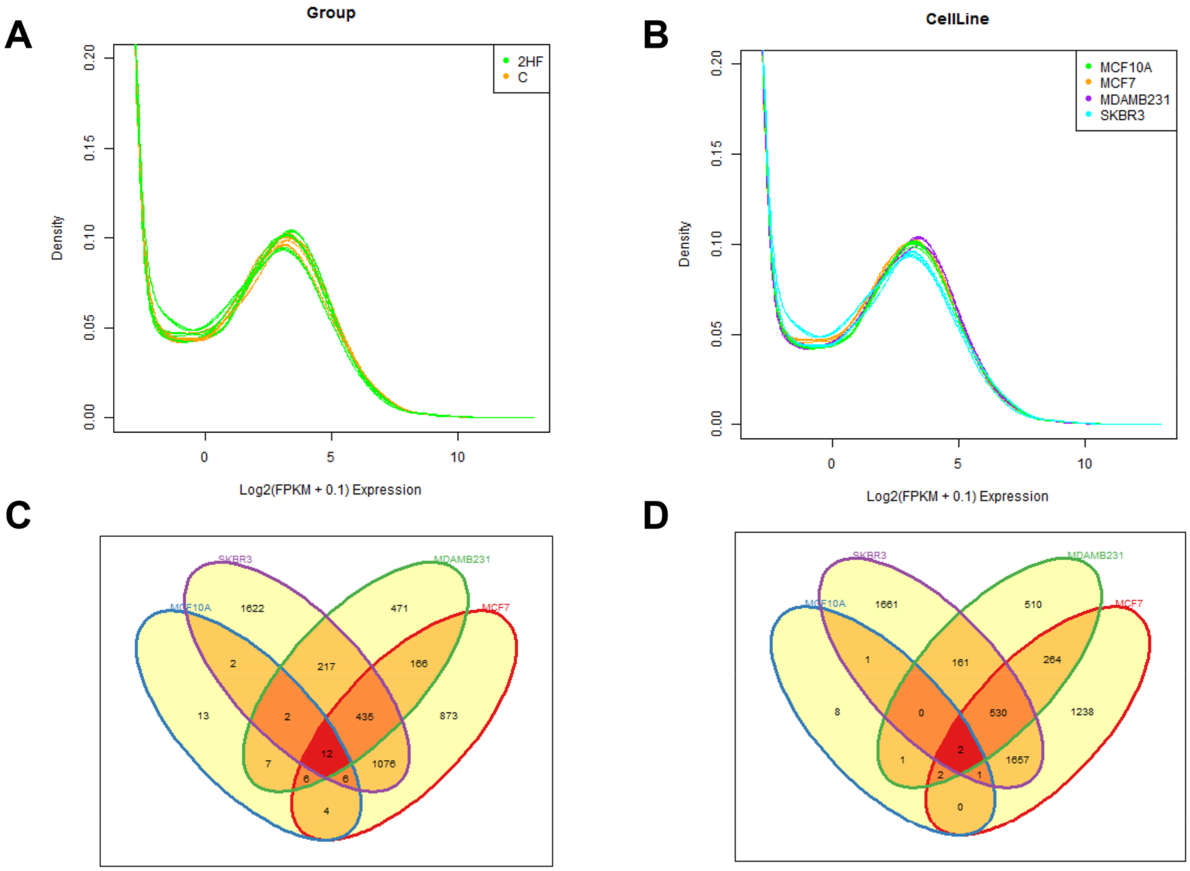

Supplementary Figure 1: (A-B) RNA-Seq Read-alignment characteristics in 2’-hydroxyflavanone (2HF) treated and control normal breast epithelial cells and MCF7, MDA-MB231 and SKBR3 breast cancer cells. Venn diagram showing the overlapping up regulated **(C)** and down regulated **(D)** genes following 2HF treatment in control normal breast epithelial cells and MCF7, MDA-MB-231 and SKBR3 breast cancer cells.

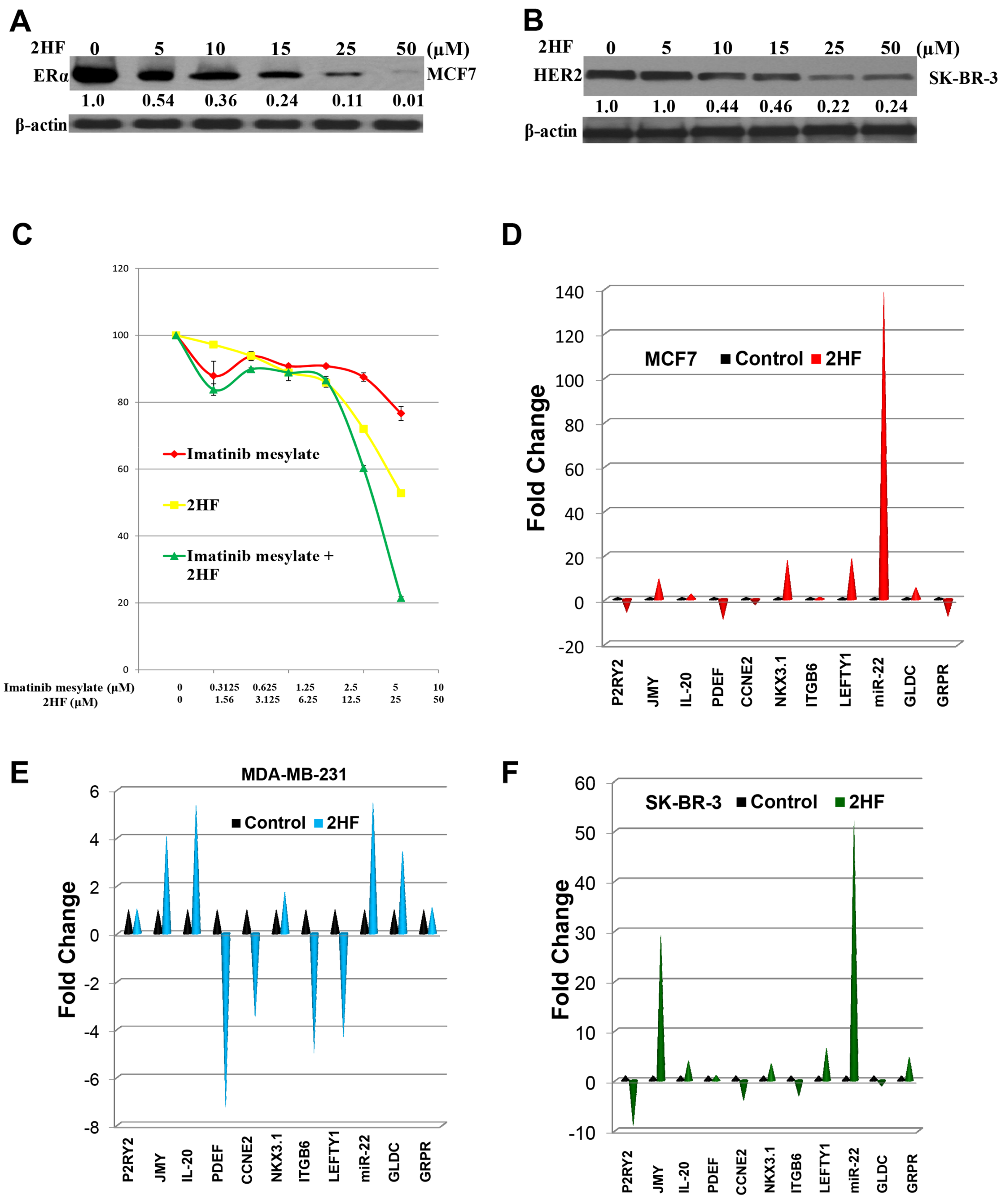

Supplementary Figure 2: Validation of key breast cancer proteins and genes regulated by 2HF. (A) Effect of 2HF on ERα expression in ER+ MCF7 cell: Aliquots of crude membrane extracts (100 µg) from control and 2HF-treated (range 5 -50 µM) MCF7 cells were applied to SDS-PAGE and subjected to Western blot analyses against anti-ERα IgG. Results were quantified by scanning densitometry. β-actin was used as an internal control. Numbers below the blots represent the fold change in the level of ERα as compared to control as determined by densitometry. **(B)** Effect of 2HF on HER2 expression in HER2+ SKBR3 cells: Aliquots of crude membrane extracts (100 µg) from control and 2HF-treated (range 5 -50 µM) SKBR3 cells were applied to SDS-PAGE and subjected to Western blot analyses against anti-HER2 IgG. Results were quantified by scanning densitometry. β-actin was used as an internal control. Numbers below the blots represent the fold change in the level of HER2 as compared to control as determined by scanning densitometry. **(C)** Effect of 2HF on imatinib mesylate/Gleevec induced inhibition: CellTiter Glo assay using 2HF alone and in combination with imatinib mesylate at 48 h post treatment in ER+ MCF7 cells. **(D-F)**, Validation of 2HF induced changes in gene expression in MCF7 (D), MDA-MB-231 (E), and SKBR3 (F) cells by qRT-PCR.

Analysis: n6_2HF_vs C_MCF10A (|fc| > 2, FDR < 0.01)

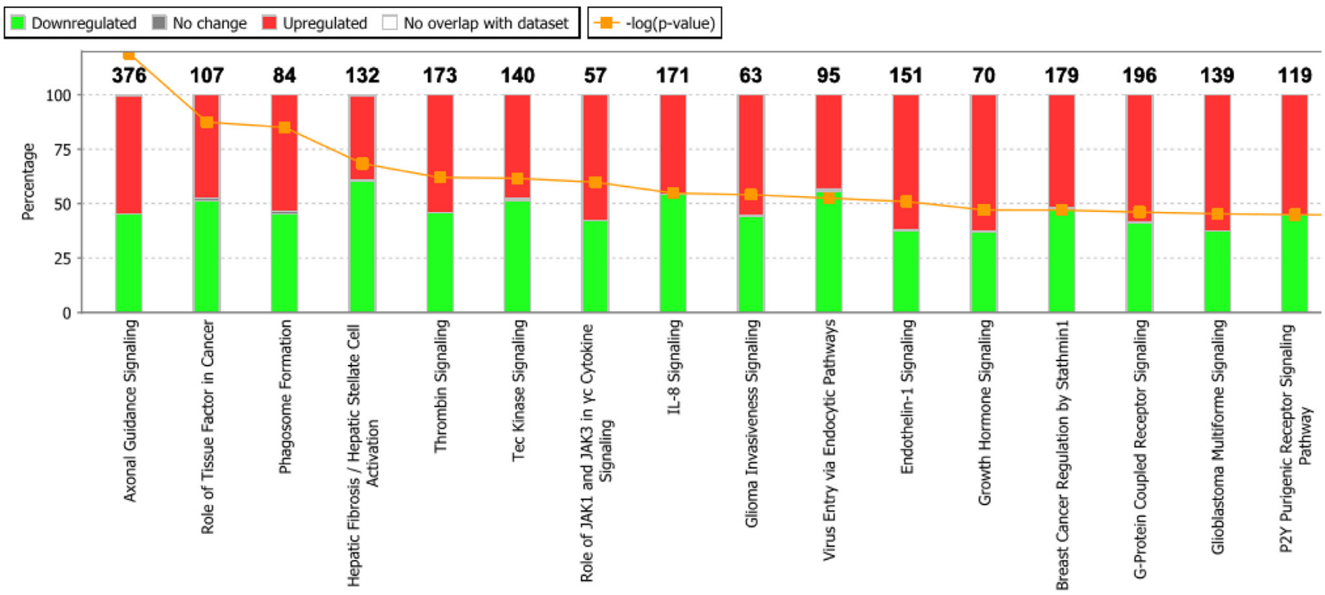

Supplementary Figure 3: IPA analyses of differentially regulated canonical pathways following 2HF treatment in MCF10A normal breast epithelial cells. Gene expression color code: Red-upregulated; Green-Downregulated.

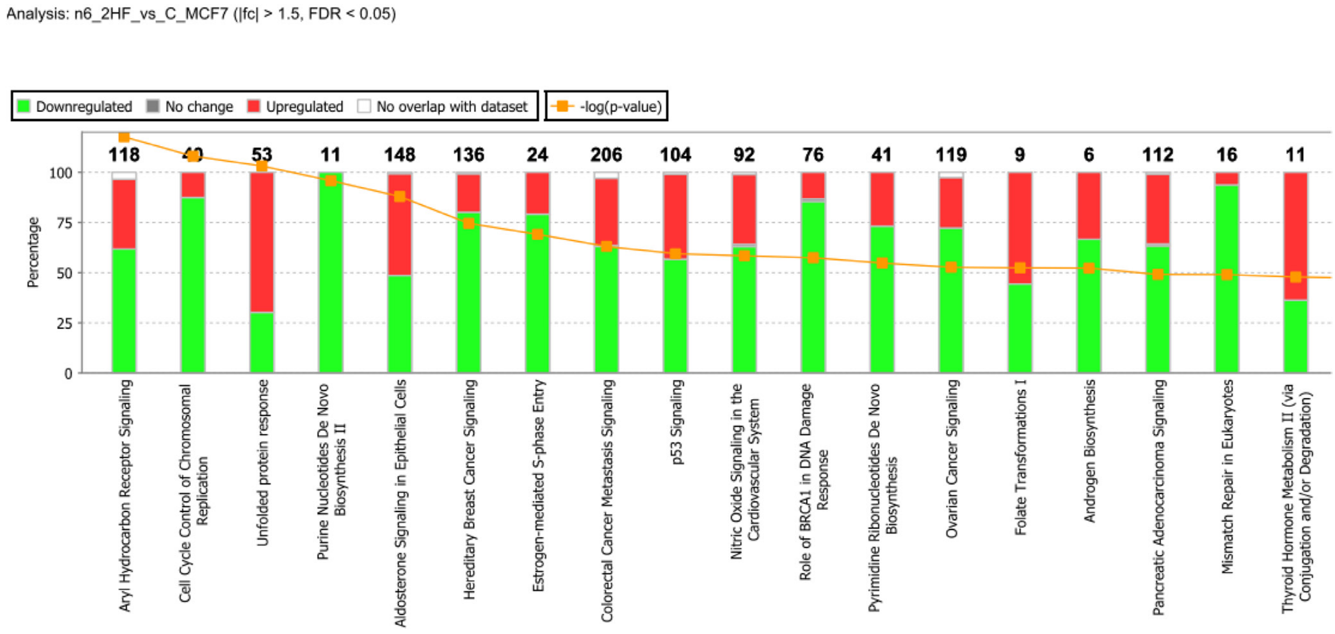

Supplementary Figure 4: IPA analyses of differentially regulated canonical pathways following 2HF treatment in MCF7 breast cancer cells. Gene expression color code: Red-upregulated; Green-Downregulated.

Analysis: n6_2HF_vs_C_MDAMB231 (|fc| > 1.5, FDR < 0.05)

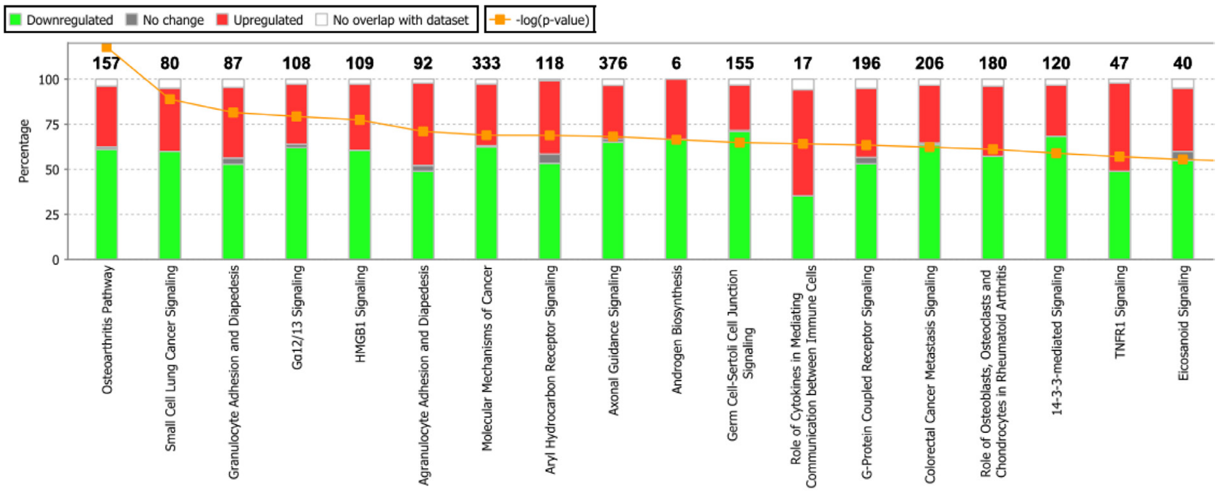

Supplementary Figure 5: IPA analyses of differentially regulated canonical pathways following 2HF treatment in MDA-MB231 breast cancer cells. Gene expression color code: Red-upregulated; Green: Downregulated.

Analysis: n6_2HF_vs_C_SKBR3 (|fc| > 1.5, FDR < 0.05)

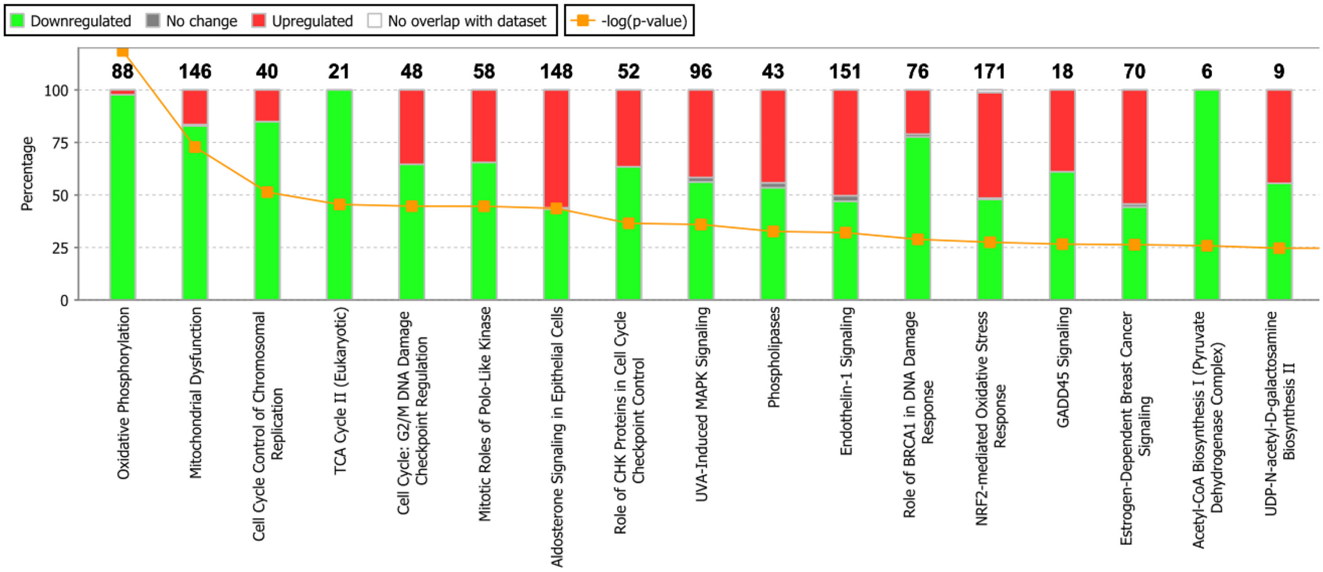

Supplementary Figure 6: IPA analyses of differentially regulated canonical pathways following 2HF treatment in SKBR3 breast cancer cells. Gene expression color code: Red-upregulated; Green-Downregulated.

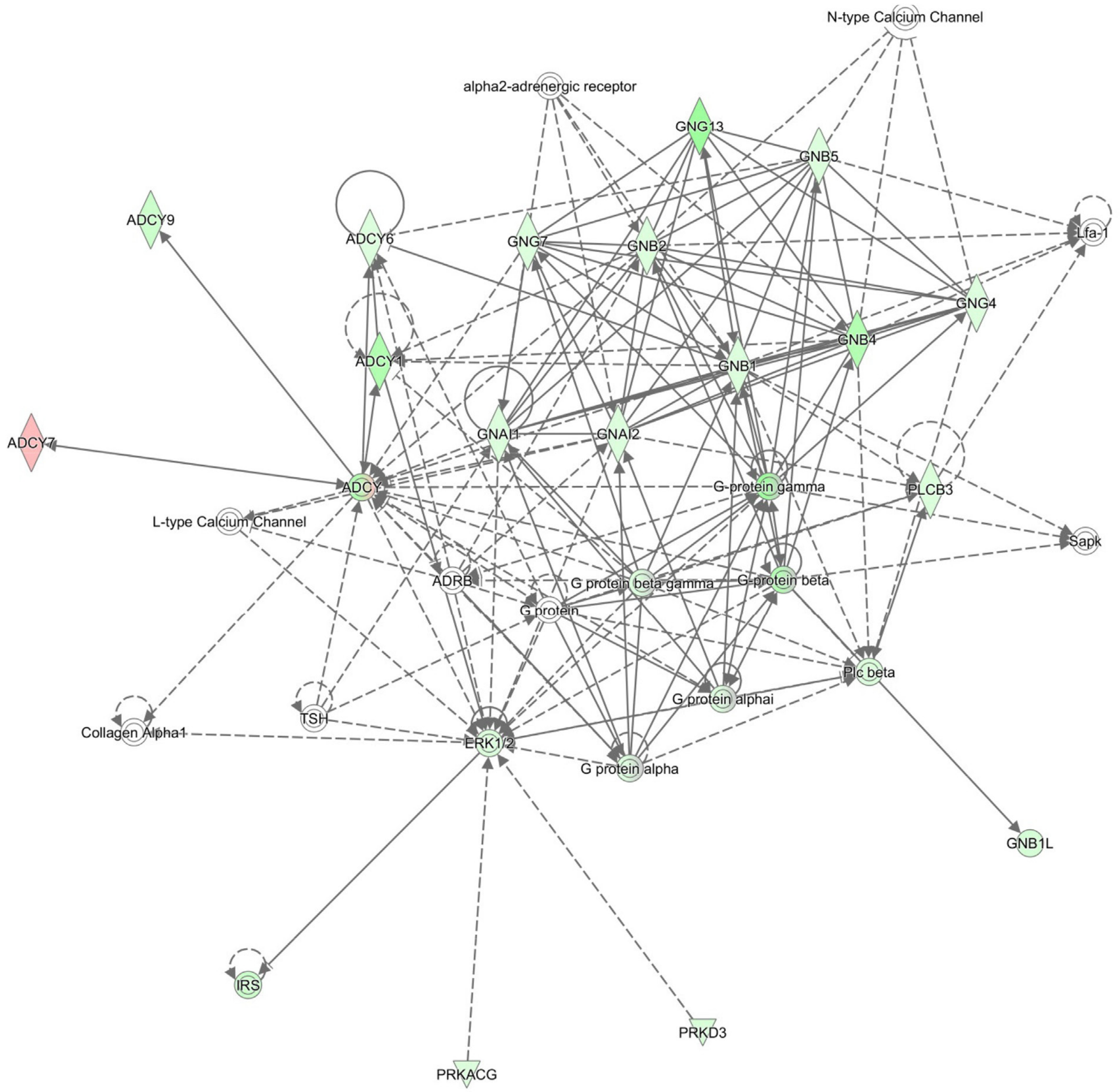

Supplementary Figure 7: IPA analyses of differentially regulated genes of P2RY2 purinergic receptor signaling network following 2HF treatment in MCF7 breast cancer cells. Gene expression color code: Red-upregulated; Green-Downregulated.

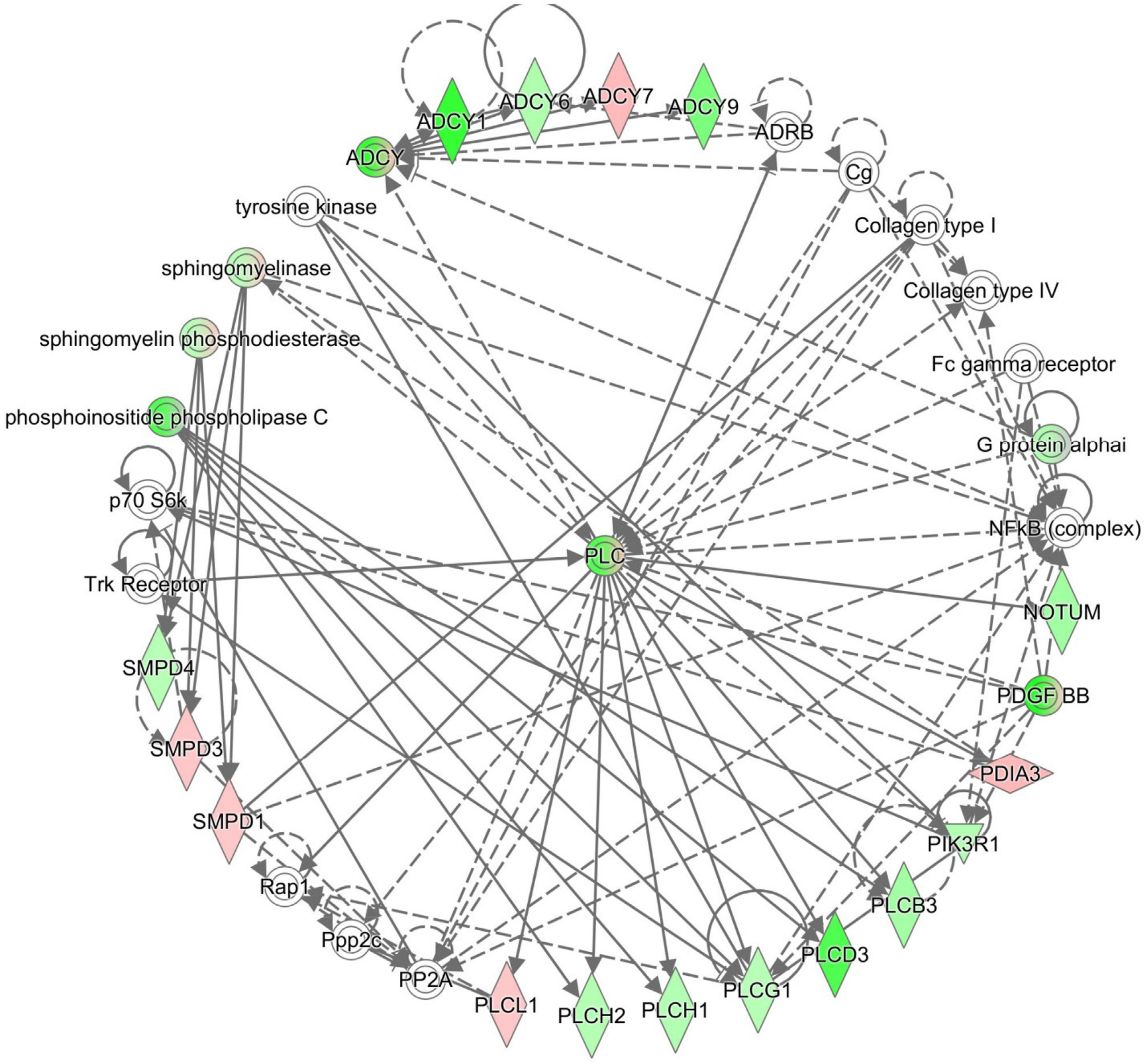

Supplementary Figure 8: IPA analyses of differentially regulated Sphingosine-1-phosphate network following 2HF treatment in MCF7 breast cancer cells. Gene expression color code: Red-upregulated; Green-Downregulated.

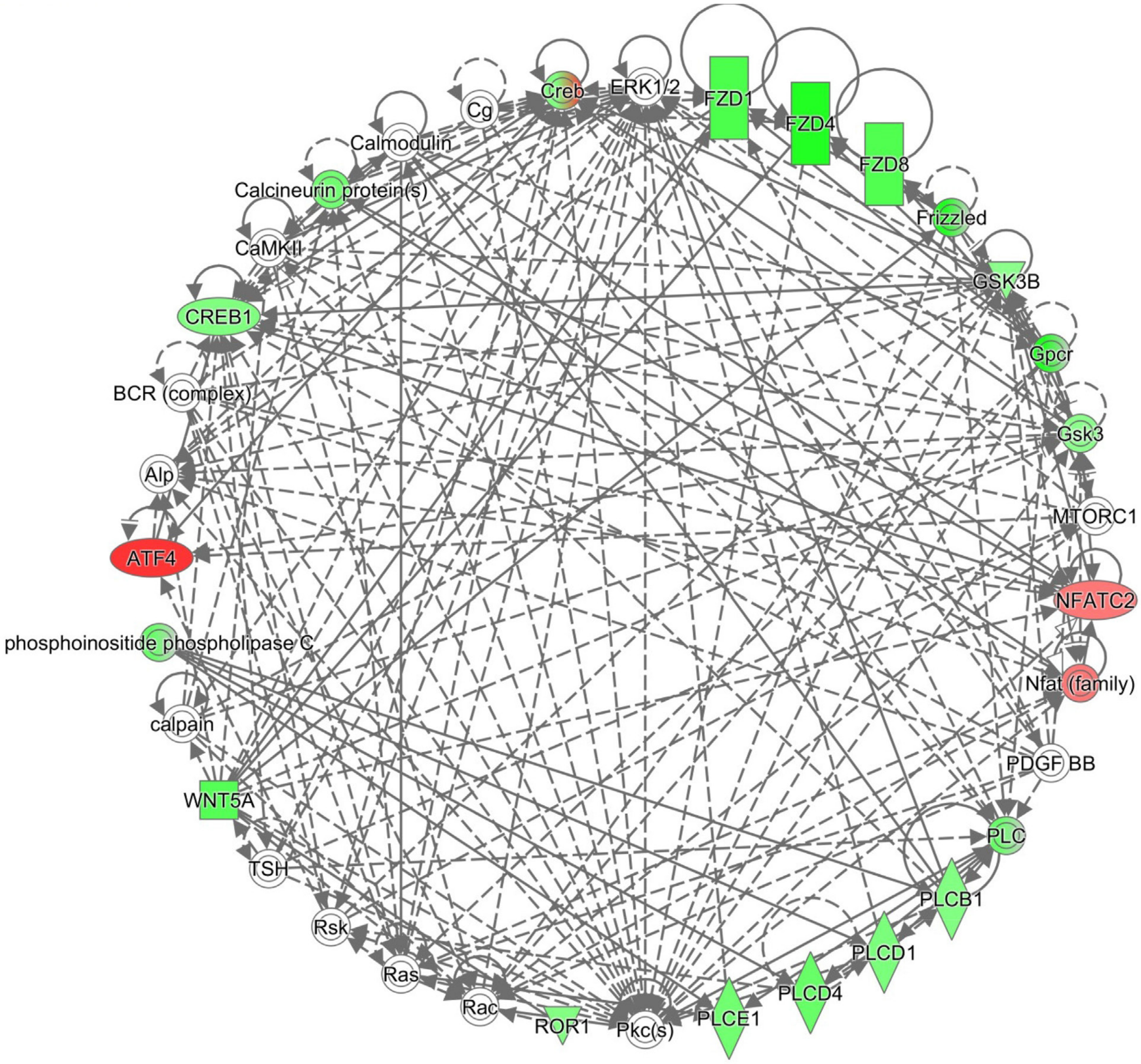

Supplementary Figure 9: IPA analyses of differentially regulated of Planar cell polarity (PCP) network following 2HF treatment in MDA-MB231 breast cancer cells. Gene expression color code: Red-upregulated; Green-Downregulated.

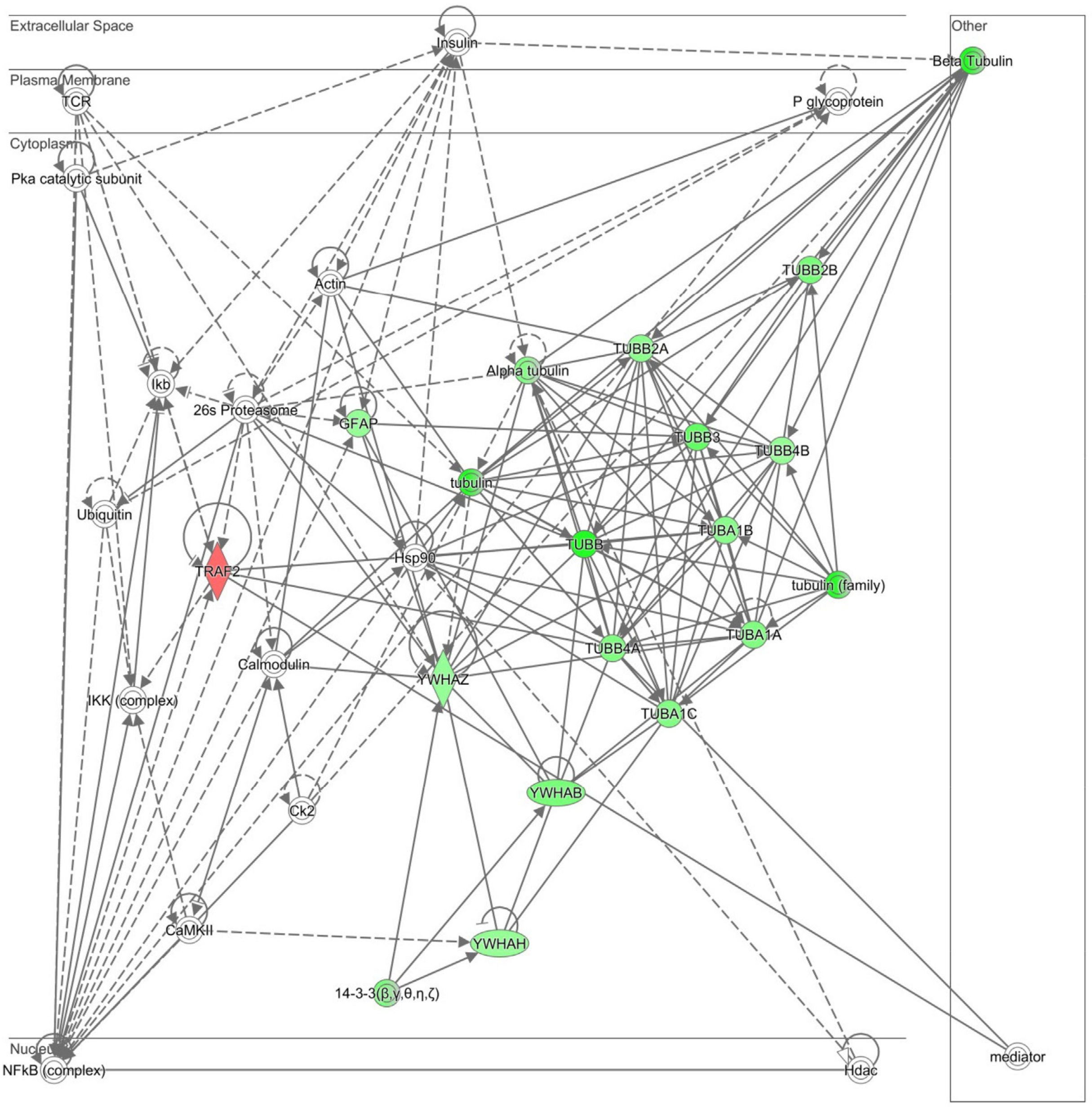

Supplementary Figure 10: IPA analyses of differentially regulated of 14-3-3 protein signaling network following 2HF treatment in MDA-MB231 breast cancer cells. Gene expression color code: Red-upregulated; Green-Downregulated.

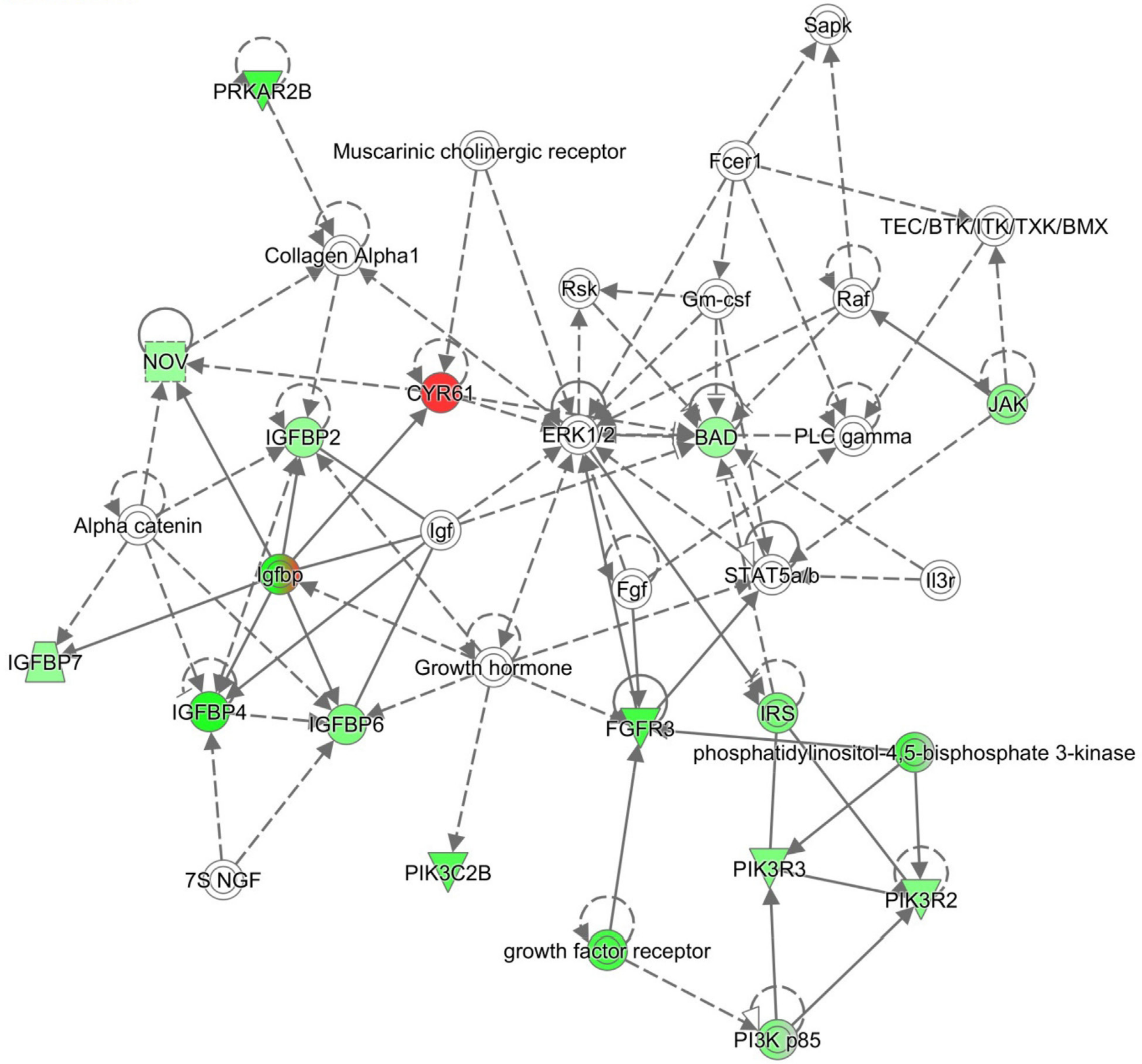

Supplementary Figure 11: IPA analyses of differentially regulated of IGF1 signaling network following 2HF treatment in MDA-MB231 breast cancer cells. Gene expression color code: Red-upregulated; Green-Downregulated.

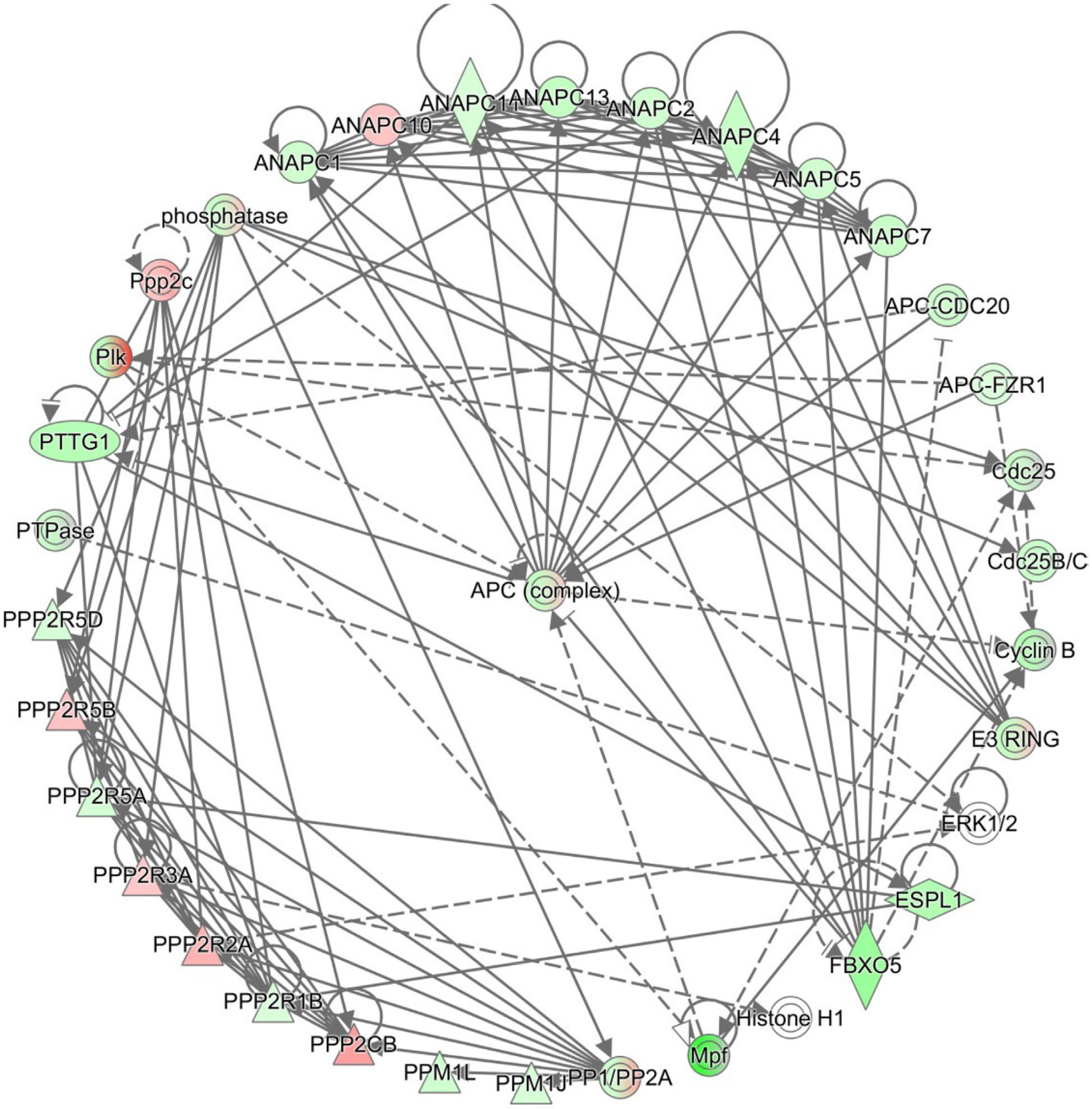

Supplementary Figure 12: IPA analyses of differentially regulated of polo-like kinase mediated mitotic activity network following 2HF treatment in SKBR3 breast cancer cells. Gene expression color code: Red-upregulated; Green-Downregulated.

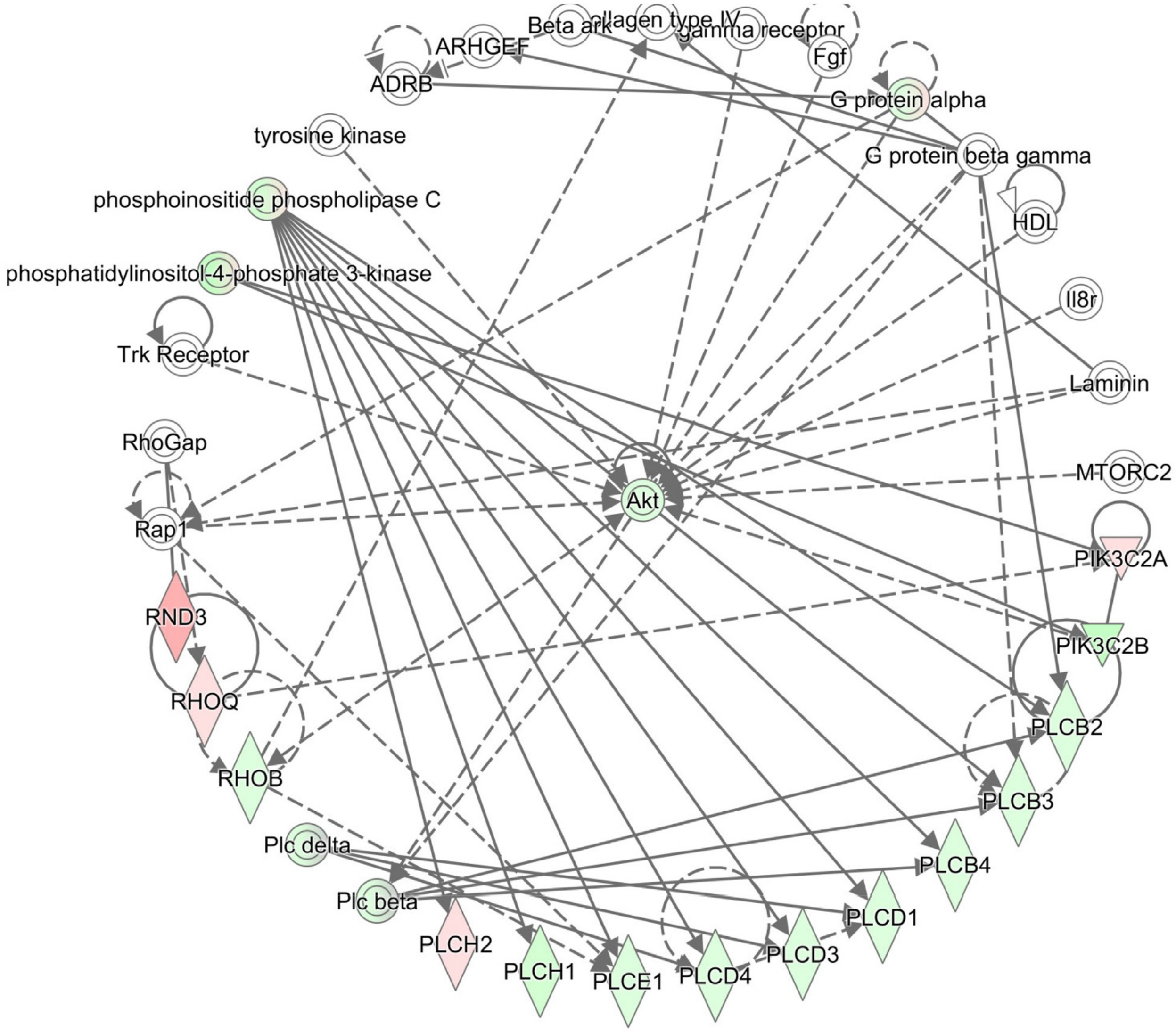

Supplementary Figure 13: IPA analyses of differentially regulated of sphingosine-1-phosphate network following 2HF treatment in SKBR3 breast cancer cells. Gene expression color code: Red-upregulated; Green-Downregulated.

Supplementary Table 1: Version of software used for analyses of results from RNA-Seq following 2HF treatment in breast cancer cells

Analysis	Software
Read Counts	htseq-count (version 0.6.0)<br>TxDb.Hsapiens.UCSC.hg19.knownGene (downloaded 6/8/2016)
QC / Normalization	R-3.3
DEG	DESeq2 (version 1.12.4)
GO	goseq (version 1.24.0)
Heatmaps	heatmap.3
IPA	Ingenuity IPA<br>Building_narnia, Date 7/27/2017

Supplementary Table 2: Read alignment statistics for RNA-Seq analyses of 2HF treated and Control breast cancer cells

Cell Line	Group	Read Count (Total)	Percent Aligned	Percent Exonic (Unique)	Transcript Coverage	Trimmed Percentage	Strand Fraction	TIN Score
MCF10A	Control	35,376,163	93.80%	86.90%	66.40%	48.40%	0.98	77
MCF10A	Control	32,978,175	93.20%	85.90%	67.00%	49.40%	0.98	77.4
MCF10A	Control	36,186,392	93.10%	86.10%	66.60%	49.20%	0.98	77.2
MCF10A	2HF	36,613,970	93.10%	85.50%	66.90%	49.00%	0.98	77.6
MCF10A	2HF	41,252,272	92.80%	84.80%	67.10%	49.20%	0.98	78
MCF10A	2HF	37,279,960	92.90%	85.30%	66.50%	49.10%	0.98	77.2
MCF7	control	25,262,006	93.70%	84.60%	67.60%	51.30%	0.97	74.3
MCF7	control	24,137,931	93.90%	85.30%	67.60%	51.10%	0.97	73.7
MCF7	control	24,028,088	93.60%	85.20%	67.20%	51.00%	0.97	72.9
MCF7	2HF	23,929,026	94.00%	85.40%	66.90%	48.70%	0.98	73.1
MCF7	2HF	24,934,218	93.80%	85.10%	67.30%	48.80%	0.98	72.7
MCF7	2HF	21,287,564	93.30%	85.20%	66.40%	48.80%	0.98	72
MDA-MB-231	control	24,707,059	95.30%	88.70%	65.80%	51.10%	0.99	72.9
MDA-MB-231	control	27,809,034	95.50%	89.10%	66.30%	51.20%	0.99	73.4
MDA-MB-231	control	28,276,448	95.90%	89.40%	66.30%	51.20%	0.99	73.8
MDA-MB-231	2HF	27,478,734	95.70%	86.70%	66.60%	51.30%	0.98	74.1
MDA-MB-231	2HF	39,269,069	96.20%	85.10%	68.20%	51.20%	0.98	75.4
MDA-MB-231	2HF	29,549,459	95.00%	85.70%	66.60%	51.60%	0.98	73.9
SKBR3	Control	36,230,390	93.00%	86.30%	66.60%	47.20%	0.97	77
SKBR3	Control	36,278,970	93.30%	86.30%	67.50%	47.10%	0.97	76.9
SKBR3	Control	36,760,026	93.00%	85.80%	66.80%	47.00%	0.97	76.9
SKBR3	2HF	38,236,714	93.80%	85.80%	71.00%	44.40%	0.98	75.9
SKBR3	2HF	37,976,299	93.40%	86.30%	70.90%	44.20%	0.98	75.4
SKBR3	2HF	42,240,169	92.90%	85.80%	71.10%	44.80%	0.98	76.3

Good alignment stats → Should be at least 70% for polyA library (both overall and exonic, within aligned reads); Good percentage of expressed genes (with RPKM > 0.1) → This is typically around 70%; Trimmed Percent reads is the percent of reads assigned to the 30th to 95th percentile of genes (similar to TMM normalization in edgeR); Usually ~50%; Strand fraction refers to the proportion of reads mapped to opposite strand of gene annotation. It is close to 0.5 for unstranded libraries. For stranded Illumina libraries, the strand of the template is the opposite of the strand of read (so, strand fraction value is close to 1). TIN score is like RIN score, but genome-wide and with range from 0 to 100; Median TIN score usually greater than 70. Significant problem if it drops below 50.

Supplementary Table 3: MCF10A_2HF vs. Control gene expression changes

See Supplementary File 1

Supplementary Table 4: MCF7_2HF vs. Control gene expression changes

See Supplementary File 1

Supplementary Table 5: MDA-MB-231_2HF vs. Control gene expression changes

See Supplementary File 1

Supplementary Table 6: SKBR3_2HF vs. Control gene expression changes

See Supplementary File 1

Supplementary Table 7: Regulation of critical genes following 2HF treatment in breast cancer cells

Symbol	gene.length.kb	Fold Change		
		MCF7	MDA-MB-231	SKBR3
AFAP1L2	5.676	-14.52	-2.41	-2.73
AKR1C1	2.823	163.14	18.64	_
AKR1C2	4.143	109.14	7.31	_
BEX1	0.859	10.56	_	_
CA9	1.543	-2.22	-7.16	-3.84
CCNE2	-7.01	-5.5		-10.48
CLIC3	0.807	-15.56	-2.48	-6.06
ERN1	3.983	8.34	1.65	8.51
GABARAPL1	2.728	63.56	2.85	24.42
GLDC	3.784	_	_	-9.71
GRPR	2.678	-10.56	_	_
IL-20	1.229	-6.73	-10.78	_
ITGB6	3.196	_	_	-7.36
JMY	9.125	4.53	1.95	6.59
KCNN4	2.231	-15.14	-1.64	-5.39
LEFTY1	2.57	_	-11	_
MIR210HG	2.301	-15.03	-2.89	-6.19
MIR22HG	2.734	27.86	2.35	7.26
NKX3-1	3.279	8.11		
NR2F1	3.194	-19.43	-5.78	_
P2RY2	8.932	-14.83	_	-39.4
SOX9	3.931	-1.72	-13.55	_
SPDEF	1.908	-7.57	-10.78	-2.68
SPRY1	3.036	-9.32	-9.78	-4.08
TMPRSS4	3.562	-13.74	_	-5.17
TP73	6.702	-6.23	-2.77	_

Supplementary Table 8: _MCF10A_2HF vs. Control_Upstream Analyses

See Supplementary File 1

Supplementary Table 9: MCF7_2HF vs. Control_ Upstream Analyses

See Supplementary File 1

Supplementary Table 10: MDA-MB-231_2HF vs.Control_Upstream Analyses

See Supplementary File 1

Supplementary Table 11: SKBR3_2HF vs. Control_Upstream Analyses

See Supplementary File 1

Supplementary Table 12: Regulation of MammaPrint genes by 2HF treatment in breast cancer cells

Symbol	gene.length.kb	Fold Change		
		MCF7	MDA-MB-231	SKBR3
KDM7A	9.251	4.17	2.55	_
ZNF385B	4.315	-12.3	-2.2	_
CCNE2	2.738	-7.01	-5.5	-10.48
MCM6	3.77	-4.96	-1.97	-4.63
DCK	2.689	-2.39	-1.77	-1.96
LPCAT1	4.076	-2.69	-1.72	-1.61
DTL	4.397	-4.35	-1.61	-5.66
GNAZ	3.205	-2.89	-1.61	-2.17
ADGRG6	7.26	-3.23	-1.59	_
ECI2	1.643	-1.74	-1.54	-2.01
MELK	2.59	-2.41	-1.51	-4.41
NMU	0.806	-3.36	-2.19	-4.26
NUSAP1	2.443	-2.39	-2.19	-6.96
EGLN1	7.227	-1.92	-2.1	-2.23
IGFBP5	6.235	-8.28	_	-25.28
TGFB3	3.431	-6.23	_	-15.14
RTN4RL1	3.162	-5.5	_	-7.01
RASSF7	1.752	-3.18	_	-3.56
SLC2A3	3.928	-3.07	_	_
OXCT1	3.759	-2.04	_	_
CDCA7L	2.951	-1.96	_	_
RFC4	1.922	-1.95	_	-2.43
CMC2	0.726	-1.72	_	-2.45
RAB6B	5.682	-1.72	-1.53	_
ALDH4A1	3.565	-1.67	_	-3.76
ECT2	4.792	-1.56	_	-3.29
GMPS	2.44	-1.55		
BBC3	2.09	3.73	_	4.14
SCUBE2	4.684	_	_	2.03
HRASLS2	0.738	_	_	-2.69
EBF4	2.893	-1.62	1.58	-1.54
EXT1	3.352	3.61	_	3.36
CENPA	1.561	_	_	-2.2
PRC1	3.641	_	_	-2.6
CDCA7	2.8	-6.11	_	-7.16
GPR180	8.866	_	_	1.59
MMP9	2.323	-2.81		
